# Comparative Effectiveness of Amiodarone Versus Lidocaine as Antiarrhythmic Therapy for Cardiac Arrest with Shockable Rhythms: A Systematic Review and Meta-Analysis

**DOI:** 10.3390/jcm15187274

**Published:** 2026-09-19

**Authors:** Faizan Ahmed, Ayesha Zulfiqar, Tooba Nihal, Maliha Khalid, M Rafiqul Islam, Fnu Abubakar, Noor ul Ain Saleem, Haris Bin Tahir, Hafsa Arshad Azam Raja, Areej Dar, Omar Kamel, Faseeh Haider, Saman Rauf, Wania Ayub, Khush Dadhania, Mobeen Zaka Haider, Yasar Sattar, Amro Taha

**Affiliations:** 1 Department of Internal Medicine, Hackensack Meridian Health, Jersey Shore University Medical Center, Neptune, NJ 07753, USA; faizan.ahmed1@hmhn.org; 2 Department of Internal Medicine, Dow University of Health and Sciences, Karachi 74200, Pakistan; 3 Department of Internal Medicine, Allama Iqbal Medical College, Lahore 54550, Pakistan; tubanihaalwork@gmail.com (T.N.); faseehhaidermd@gmail.com (F.H.); 4 Department of Internal Medicine, Jinnah Sindh Medical University, Karachi 75500, Pakistan; malihakhalid2002@gmail.com; 5 Department of Internal Medicine, Shaheed Suhrawardy Medical College Hospital, Dhaka 1207, Bangladesh; islamrafiqulmedicine@gmail.com; 6 Department of Internal Medicine, Sanford School of Medicine, University of South Dakota, Sioux Falls, SD 57107, USA; ab.abubakar@usd.edu; 7 Department of Internal Medicine, FMH College of Medicine and Dentistry, Lahore 54000, Pakistan; 493noorulain@gmail.com; 8 Department of Internal Medicine, Lahore General Hospital, Lahore 54000, Pakistan; harristahirchh@gmail.com; 9 Department of Internal Medicine, Rawalpindi Medical University, Rawalpindi 46000, Pakistan; hafsaazamrajput@outlook.com; 10 Department of Internal Medicine, Shaikh Khalifa Bin Zayed Al-Nahyan Medical and Dental College, Lahore 54600, Pakistan; areej.dar6@gmail.com; 11 Department of Physical Therapy, South Valley University, Qena 83523, Egypt; 411996208@sohag2.moe.edu.eg; 12 Department of Internal Medicine, Fatima Jinnah Medical University, Lahore 54000, Pakistan; samanrauf9393@gmail.com; 13 School of Medicine, St. George’s University, St. George’s P.O. Box 7, Grenada; 14 Department of Cardiology, Texas Tech University Health Sciences Center, Lubbock, TX 79430, USA; khushdadhania99@gmail.com; 15 Division of Cardiology, West Virginia University, Morgantown, WV 26506, USA; mobeenzaka@gmail.com (M.Z.H.); taha61591@gmail.com (A.T.); 16 Department of Interventional Cardiology, West Virginia University, Morgantown, WV 26506, USA; mdyasarsattar@gmail.com

**Keywords:** cardiac arrest, shockable rhythms, amiodarone, lidocaine, antiarrhythmic therapy

## Abstract

**Background:** Amiodarone and lidocaine are antiarrhythmic agents recommended for advanced cardiac life support in shockable ventricular arrhythmias during cardiac arrest, yet their comparative effectiveness in in-hospital (IHCA) and out-of-hospital cardiac arrest (OHCA) remains uncertain. **Methods:** From 930 records identified in PubMed, ScienceDirect, Embase, and Cochrane Library, 14 studies were included. Outcomes were analyzed using random-effects meta-analysis in R with IHCA and OHCA subgroup analyses. Bayesian meta-analysis using the bayesmeta package assessed result credibility. **Results:** Amiodarone did not improve return of spontaneous circulation (R0SC) compared with lidocaine (Risk ratio (RR) 0.96, *p* = 0.40). In IHCA, amiodarone was associated with lower survival to hospital discharge (RR 0.88, *p* < 0.0001), lower 24 h survival (RR 0.93, *p* < 0.0001), worse neurological recovery (RR 0.83, *p* < 0.0001), and longer arrest duration (Mean difference (MD) 7.46 min, *p* = 0.002). Termination of shockable rhythms favored lidocaine only after sensitivity analysis (RR 0.51, *p* = 0.02). In OHCA, amiodarone did not improve survival to discharge (RR 0.93, *p* = 0.44), while return of spontaneous circulation favored lidocaine after sensitivity analysis (RR 0.88, *p* = 0.002). No consistent benefit was observed across outcomes. Bayesian analysis showed overlapping credible intervals and low probabilities of benefit, indicating no clear superiority. **Conclusions:** No survival difference was observed between amiodarone and lidocaine arrest. Although lidocaine showed more favorable in-hospital outcomes, long-term survival was similar, suggesting agents may be selected based on availability and practice.

## 1. Introduction

Cardiac arrest remains a leading cause of death worldwide, with an estimated annual incidence of approximately 80–100 per 100,000 populations for out-of-hospital cardiac arrest (OHCA) and 1–5 per 1000 hospital admissions for in-hospital cardiac arrest [1]. The overall survival rate to hospital discharge is still low, frequently less than 10% for OHCA and roughly 20–25% for in-hospital cardiac arrest (IHCA), despite improvements in cardiopulmonary resuscitation (CPR) and post-resuscitation care [2]. Critical interventions that have been demonstrated to greatly improve outcomes include early detection, timely CPR initiation, and rapid defibrillation, especially for cardiac arrests presenting with shockable rhythms like ventricular fibrillation (VF) and pulseless ventricular tachycardia (pVT) [3]. On the other hand, non-shockable rhythms, such as asystole and pulseless electrical activity (PEA), are linked to worse outcomes and little survival advantage from defibrillatory or anti-arrhythmic treatments. Despite improved rates of return of spontaneous circulation (ROSC) and neurologically intact survival among patients presenting with shockable rhythms, overall resuscitation outcomes remain less than ideal, emphasizing the continued necessity for refinement of advanced life support techniques [4]. Anti-arrhythmics, which support membrane stability and increase the likelihood of conversion, are advised in shock-refractory ventricular fibrillation (VF) or pulseless ventricular tachycardia (pVT) [5]. Amiodarone is a Class III anti-arrhythmic that helps suppress recurrent VF/VT by blocking sodium, potassium, and calcium channels, prolonging repolarization, and having anti-adrenergic effects [6]. With a shorter half-life and fewer extracardiac effects, lidocaine, a Class Ib sodium-channel blocker, quickly reduces ventricular automaticity, especially in ischemic myocardium. Pharmacologic distinctions inform clinical decision-making, as lidocaine is characterized by rapid onset and titratability but comparatively limited durability and increased toxicity, while amiodarone provides more comprehensive arrhythmia suppression with an increased risk of hypotension and QT interval prolongation [7].

There is conflicting evidence from observational and randomized studies comparing lidocaine and amiodarone in shock-refractory VF/pVT. In out-of-hospital cardiac arrest, preliminary data showed that amiodarone was associated with a higher survival rate to hospital admission when compared to lidocaine, indicating an early benefit [8]. Nevertheless, the large Amiodarone, Lidocaine, or Placebo Study (ALPS) trial contradicted existing data by finding no discernible difference in favorable neurologic outcomes or survival to discharge between amiodarone, lidocaine, and placebo [9]. Some conflicting trends can be seen in more recent observational data with regard to in-hospital cardiac arrest and anti-arrhythmic choice [10].

The evidence comparing amiodarone and lidocaine in shockable cardiac arrest is still insufficient and inconsistent despite a large number of observational and randomized studies. Furthermore, there is a significant knowledge gap regarding in-hospital cardiac arrest, during which patient characteristics, arrest causes, monitoring capabilities, and treatment timelines vary significantly. To date, neither medication has been directly assessed in IHCA and OHCA settings in any thorough meta-analysis. This study aims to integrate the available evidence to clarify the comparative effectiveness of amiodarone and lidocaine in shock-refractory VF/pVT across IHCA and OHCA settings, synthesizing resuscitation efficacy and clinically meaningful survival outcomes to inform evidence-based resuscitation practice and guideline refinement.

## 2. Methods

### 2.1. Protocol Registration

This systematic review and meta-analysis was conducted in accordance with the Preferred Reporting Items for Systematic Reviews and Meta-Analyses (PRISMA) guidelines [11] to ensure transparency and methodological rigor. The study protocol was prospectively registered on the International Prospective Register of Systematic Reviews (PROSPERO) database (Registration ID: CRD420251276136) prior to data extraction. PRISMA checklist is provided in Supplementary Table S1.

### 2.2. Search Strategy

A comprehensive search strategy was developed and applied across major electronic databases, including PubMed, ScienceDirect, Embase, and the Cochrane Library, covering all available studies up to September 2025. The database search was conducted by two reviewers individually, with no language filters applied during the initial database searches, and potentially eligible non-English-language records were therefore retrieved. During the screening and eligibility assessment, non-English-language studies were excluded because reliable data extraction could not be performed from the available reports. Grey literature sources, including conference abstracts, dissertations, preprints, and trial registries, were not systematically searched.

The search strategy incorporated Medical Subject Headings (MeSH) terms to identify studies related to cardiac arrest and anti-arrhythmic interventions. The following search string was used in PubMed and adapted for other databases: “(“Heart Arrest”[Mesh] OR “Out-of-Hospital Cardiac Arrest”[Mesh] OR “Ventricular Fibrillation”[Mesh] OR “Tachycardia, Ventricular”[Mesh]) AND (“Amiodarone”[Mesh] OR “Amiodarone Hydrochloride”[Mesh] OR “Lidocaine”[Mesh]) AND (“Emergency Medical Services”[Mesh] OR “Hospitals”[Mesh])”. The detailed search strategy for the databases is provided in Supplementary Table S2.

### 2.3. Inclusion and Exclusion Criteria

We included randomized controlled trials (RCTs) and prospective or retrospective cohort studies directly comparing amiodarone with lidocaine in eligible cardiac-arrest populations. Studies were excluded if they did not report relevant outcomes or were published as abstracts, commentaries, letters to the editor, review articles, study protocols, book chapters, or clinical guidelines. Case series and case reports were excluded because they lacked an appropriate comparative group. Case–control studies were excluded to maintain consistency in study design and effect estimation across the included comparative cohorts. Secondary and post hoc analyses were excluded to avoid duplicate inclusion of study populations and to maintain focus on the primary comparative evidence. Non-English-language publications identified during the screening process were excluded because reliable data extraction could not be performed from the available reports. This restriction may have introduced language bias and potentially resulted in the exclusion of relevant evidence published in other languages.

Two reviewers independently screened all titles and abstracts to assess relevance, followed by a detailed full-text review of potentially eligible studies. Any disagreements were resolved through discussion or consultation with a third reviewer. The inclusion criteria were based on the Population, Intervention, Comparator, and Outcome (PICO) framework, focusing on studies involving patients who experienced in-hospital or out-of-hospital cardiac arrest due to shockable rhythms such as ventricular fibrillation or pulseless ventricular tachycardia (Population). The intervention of interest was the administration of amiodarone, compared with lidocaine as the comparator. Consequently, the final analysis was restricted to full-text, peer-reviewed articles published in English.

### 2.4. Data Extraction and Management

Data were extracted using a standardized form, capturing details such as study design, participant characteristics, treatment protocols, and outcomes. Two authors independently collected this data to minimize bias. Discrepancies were resolved through discussion and agreement. All data were compiled and organized in Microsoft Excel for analysis.

### 2.5. Outcomes of Interest

The primary outcomes assessed across the included studies were Return of Spontaneous Circulation (ROSC), Survival to Hospital Discharge, 24 h Survival, and Favourable Neurological Outcome. Secondary outcomes included Termination of Ventricular Fibrillation/Pulseless Ventricular Tachycardia, Duration of Cardiac Arrest, and Survival to intensive care unit (ICU) Admission in the out-of-hospital cardiac arrest subgroup.

### 2.6. Quality Assessment & Publication Bias

Each of the RCTs was evaluated using the Cochrane Risk of Bias 2.0 (RoB 2) tool and Cohorts using the ROBINS-1 (2016 version), assessing factors like randomization process, adherence to interventions, missing outcome data, measurement reliability, and selective reporting [12,13]. Publication bias was evaluated to assess potential small-study effects and reporting bias. Doi (Deviation from Optimal Intervention) plots and the Luis Furuya-Kanamori (LFK) index were visually inspected for asymmetry in effect size distribution. Symmetry in the Doi plot and non-significant LFK test results were interpreted as providing no evidence of substantial funnel-plot asymmetry; however, these assessments cannot definitively confirm or exclude publication bias.

### 2.7. Certainty of Evidence

Certainty of evidence was assessed for each outcome using the Grading of Recommendations Assessment, Development and Evaluation (GRADE) framework across five domains: risk of bias, inconsistency, indirectness, imprecision, and publication bias. Risk of bias was informed by RoB 2 for RCTs and Risk Of Bias In Non-randomized Studies–of Interventions (ROBINS-I) for observational studies. Inconsistency was assessed using I^2^ and sensitivity analyses, while imprecision was judged based on the 95% CI and available information. Publication bias was evaluated using Doi plots and the LFK index when sufficient studies were available. Consistent with GRADE guidance for reviews applying ROBINS-I, all bodies of evidence were started at high certainty, with confounding captured within the risk-of-bias domain rather than the starting level. Overall certainty was rated as high, moderate, low, or very low; the design of the contributing studies is reported for each outcome, and the reason for every downgrade, together with the criteria considered for rating up, is given in Supplementary Table S3.

### 2.8. Statistical Analysis

The meta-analysis was conducted using R software (version 4.5.1) with the meta package (version 6.2-1). For each outcome, pooled risk ratios (RRs) and mean differences (MDs) with corresponding 95% confidence intervals (CIs) were calculated using appropriate meta-analytic models. Forest plots were generated to present study-specific and pooled estimates across clinical settings (in-hospital and out-of-hospital cardiac arrest) and outcomes. Heterogeneity among the included studies was assessed using Cochran’s Q statistic and the I^2^ index. I^2^ values below 25%, between 25% and 50%, or above 50% indicated low, moderate, or high heterogeneity, respectively [14]. A *p* value of <0.05 was always considered significant. Cochran’s Q statistic was used to assess the statistical significance of heterogeneity. The between-study variance was also examined using Tau-squared (τ^2^). The stability of the pooled estimates was assessed through a leave-one-out analysis, where each study was sequentially removed, and the remaining dataset was re-analyzed to ensure that no single study unduly influenced the aggregated effect sizes. Subgroup analyses were performed according to clinical setting (in-hospital versus out-of-hospital cardiac arrest) and individual outcomes to explore potential sources of heterogeneity and compare pooled effects.

### 2.9. Bayesian Analysis

Bayesian random-effects meta-analyses conducted on the relative (risk ratio [RR]) for binary outcomes, and as mean differences (MDs) for continuous outcomes. A continuity correction of 0.5 was applied when required. The primary model used a half-normal (0.5) prior for between-study heterogeneity (τ), with results expressed as posterior medians and 95% credible intervals (CrIs); heterogeneity was summarized by tau (τ). Prediction intervals were also derived. Model adequacy assessed by convergence diagnostics, posterior predictive checks, posterior predictive *p*-values, and Pareto-smoothed importance-sampling leave-one-out cross-validation (PSIS-LOO). Sensitivity analyses using alternative priors (half-normal [1.0], half-Cauchy [0.5], half-Cauchy [1.0]) and leave-one-out re-estimations tested robustness. All analyses were performed in R (v 4.5.1) using the meta and bayesmeta R package version 3.5.

## 3. Results

### 3.1. Literature Search

**Figure 1:**
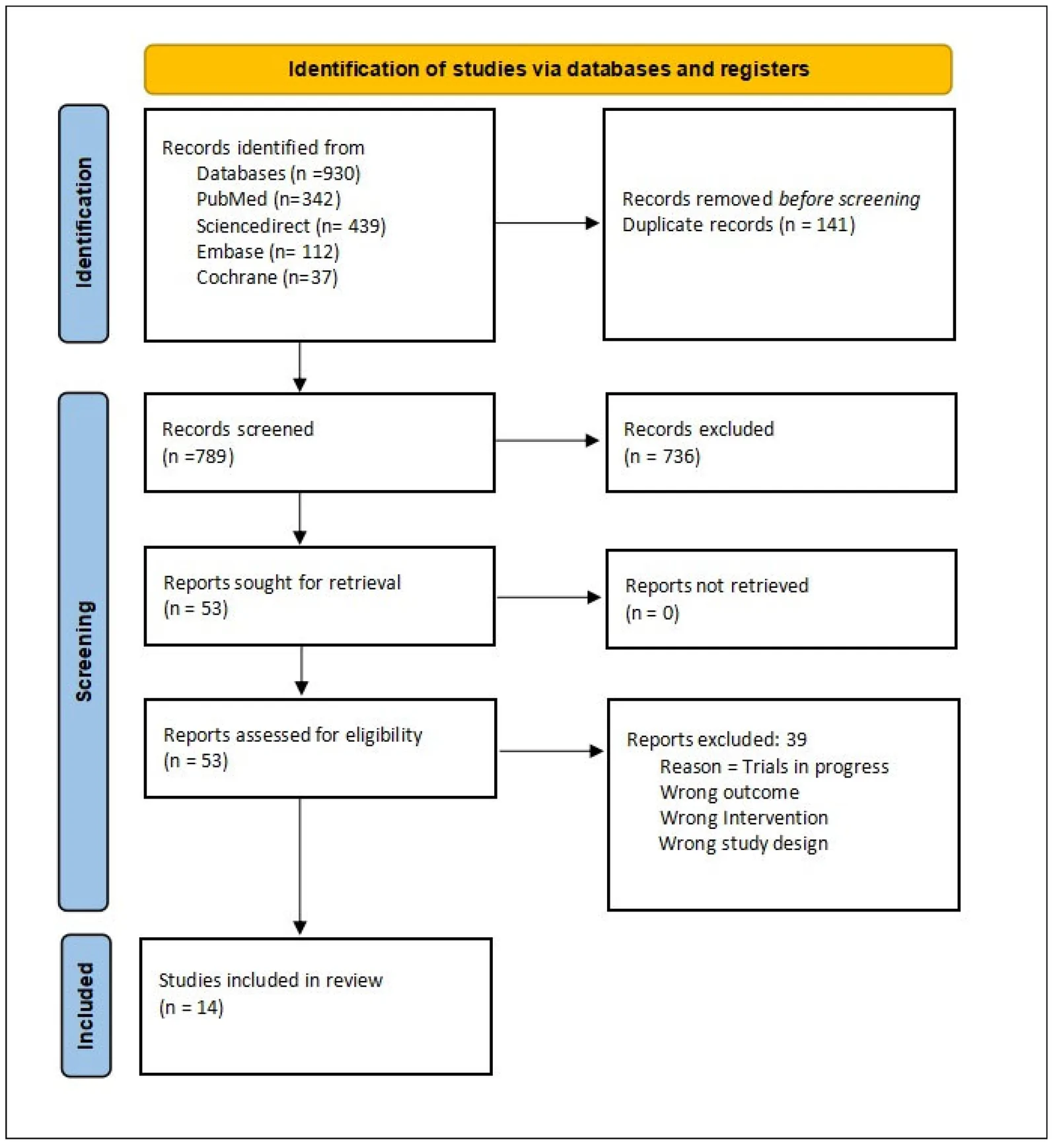
Preferred Reporting Items for Systematic Reviews and Meta-Analyses (PRISMA) flowchart illustrating the selection process.

After a meticulous literature search, 930 studies were obtained. 141 duplicates were removed. Primary screening was conducted for the subsequent studies, focusing on their titles and abstracts. Secondary screening of the remaining 53 studies was done through full-text review after excluding 736 studies. 14 studies, meeting the eligibility criteria, were included in our systematic review and meta-analysis [8,9,10,15,16,17,18,19,20,21,22,23,24,25]. The detailed screening process is set forth in the PRISMA flowchart Figure 1.

### 3.2. Baseline Characteristics

Published between 2002 and 2025, studies included 2 RCTs, 2 prospective, and 10 retrospective observational cohorts. Among those, seven studies were conducted in the US [9,10,16,18,20,21,23], while the rest were conducted elsewhere. A total of 50,773 participants were included, 15,806 patients experienced cardiac arrest in the in-hospital setting, and 34,967 patients experienced cardiac arrest in the out-of-hospital setting. The intervention group comprised 39,506 participants who received amiodarone via intravenous (IV) or intraosseous (IO) route, while the control group had 11,267 participants who received lidocaine via IV or IO route. The mean age of the participants was 64.8 years. In most studies, males were predominant. Hypotension was a common factor among 19% participants observed in 4 studies [1,4,7,8]. Detailed baseline and study characteristics are given in Table 1.

### 3.3. Quality Assessment

Two of the included studies were RCTs. Risk of bias assessment using the ROB 2.0 tool indicated that Dorian et al., 2002 and Kudenchuk et al., 2016 [8,9] demonstrated low risk of bias in all domains, which reflects the validity of the evidence. The rest of the twelve studies were observational cohorts, so ROBINS-I tool was used for quality appraisal. Only Huang et al., 2017 showed low risk overall [25]. All studies except Suzuki et al., 2016 and Smida et al., 2025 had confounders [17,23]. Six studies [15,16,18,20,21,26] with serious risk in D1 along with some concerns in most of the domains were rated to have serious risk of bias overall. Remaining five studies [17,19,22,23,24] showed moderate risk overall. A detailed assessment of the risk of bias in both study designs is outlined in the Supplementary Figures S1 and S2.

### 3.4. Outcomes

A summary of pooled effect estimates, heterogeneity, and sensitivity analyses for all outcomes is presented in Table 2, while Supplementary Table S4 summarizes study-level clinical and resuscitation characteristics that may influence treatment effects, including arrest setting, antiarrhythmic timing, shock burden, witnessed status, bystander CPR, epinephrine use, and baseline severity. These additional data are provided to facilitate more appropriate interpretation of the pooled findings, as variability in these factors may have contributed to the observed heterogeneity.

#### 3.4.1. Primary Outcomes

##### Return of Spontaneous Circulation (ROSC)

This outcome was discussed across 10 studies with 16,605 participants in the intervention group and 9334 in the control group. Among these, six studies reported outcomes for in-hospital cardiac arrest and four studies reported outcomes for out-of-hospital cardiac arrest.

1. Overall Analysis

**Figure 2:**
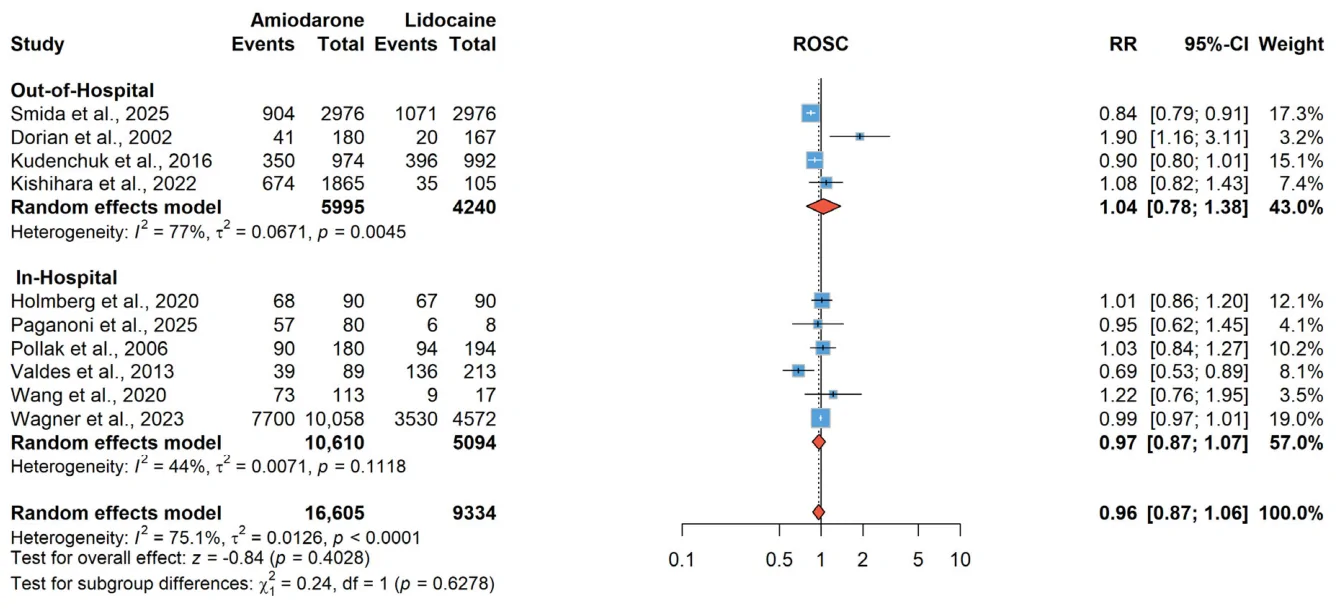
Forest Plot of Subgroup Analysis for Return of Spontaneous Circulation (ROSC) [8,9,10,15,16,19,20,21,23,24]. Effect estimates compare amiodarone with lidocaine; RR < 1 favors lidocaine, whereas RR > 1 favors amiodarone. Squares indicate study-specific estimates, with their size reflecting study weight; horizontal lines represent 95% confidence intervals, and diamonds indicate pooled estimates.

When results were compiled for analysis across all ten studies, comparison of amiodarone to lidocaine showed no significant effect on resuscitation and ROSC (RR = 0.96, 95% CI = 0.87–1.06, *p*-value = 0.40). Heterogeneity was considerable (I^2^ = 75%), suggesting variability among included studies (Figure 2).

2. ROSC in Out-of-Hospital Cardiac Arrest (OHCA)

In the OHCA subgroup analysis, statistical analysis of four studies revealed a non-significant improvement in ROSC among patients receiving amiodarone (RR = 1.04, 95% CI = 0.78–1.38, *p*-value = 0.78). Heterogeneity was relatively high (I^2^ = 77%) (Supplementary Figure S3). Sensitivity analysis (leave-one-out method) indicated that exclusion of Dorian et al., 2002 [8] substantially altered the pooled effect size rendering it significant (RR= 0.88, 95% CI = 0.81–0.95, *p*-value = 0.0017) and making heterogeneity moderate (41.8%) (Supplementary Figure S4).

3. ROSC in In-Hospital Cardiac Arrest (IHCA)

For the IHCA subgroup, the integrated analysis of six studies demonstrated no statistically significant difference in the ROSC between amiodarone and control groups (RR= 0.97, 95% CI = 0.87–1.07, *p*-value = 0.11) (Supplementary Figure S5). Moderate heterogeneity was observed (I^2^ = 44%). Sensitivity analysis (leave-one-out method) confirmed the stability of the heterogeneity and results (Supplementary Figure S6).

##### Survival to Hospital Discharge

A total of 13 studies were included in the analysis evaluating survival to hospital discharge among patients treated with amiodarone versus lidocaine during cardiac arrest. Further stratification was performed according to the setting in which cardiac arrest occurred, in-hospital cardiac arrest and out-of-hospital cardiac arrest, to explore potential subgroup differences among the included studies.

1. Survival to Hospital Discharge in In-Hospital Cardiac Arrest (IHCA)

**Figure 3:**
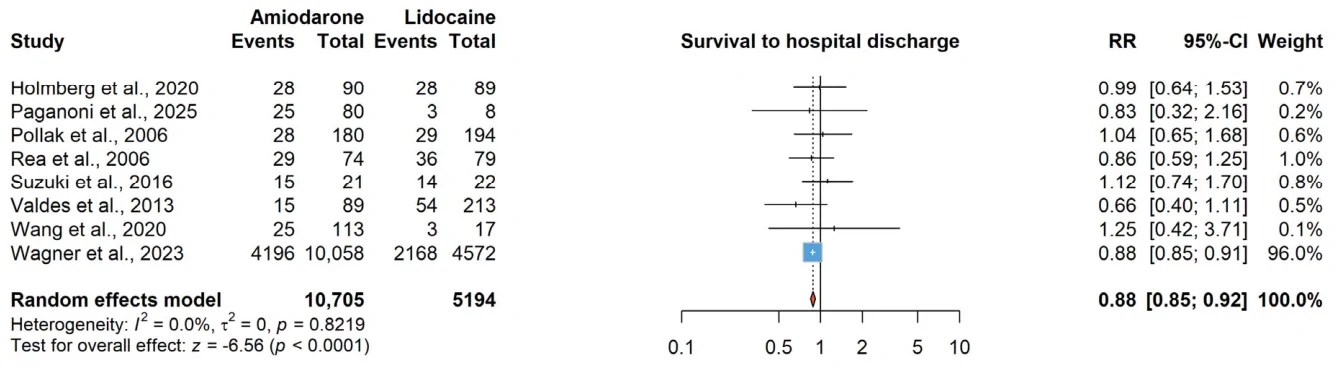
Forest Plot of Survival to Hospital Discharge in In-Hospital Cardiac Arrest (IHCA) [10,15,16,17,18,19,20,21]. Effect estimates compare amiodarone with lidocaine; RR < 1 favors lidocaine, whereas RR > 1 favors amiodarone. Squares indicate study-specific estimates, with their size reflecting study weight; horizontal lines represent 95% confidence intervals, and diamonds indicate pooled estimates.

Subgroup analysis included eight studies encompassing 15,899 patients (amiodarone *n* = 10,705; lidocaine *n* = 5194) and reporting outcomes for IHCA. The pooled estimate demonstrated a substantial difference in survival to hospital discharge among patients on the two drugs (RR = 0.88, 95% CI = 0.85–0.92, *p*-value < 0.0001). An I^2^ value of 0% suggested robust results with no observed heterogeneity (Figure 3). Sensitivity analysis (leave-one-out method) confirmed the consistency of the results across all the studies except with the exclusion of Wagner et al., 2023 [10] (RR = 0.94, 95% CI = 0.78–1.13, *p*-value = 0.49) (Supplementary Figure S7).

2. Survival to Hospital Discharge in Out-of-Hospital Cardiac Arrest (OHCA)

A total of 11,389 amiodarone and 6009 lidocaine patients were analyzed for OHCA across five studies. When combined within the framework of a random-events model, no statistically significant difference in survival to discharge (RR = 0.93, 95% CI = 0.76–1.13, *p*-value = 0.44) was observed and the results were comparable. However, heterogeneity was substantial (I^2^ = 85%), driven primarily by variability in study populations and resuscitation setting (Supplementary Figure S8). Sensitivity analysis (leave-one-out method) significantly reduced the heterogeneity (I^2^ = 0%) but the results were consistently non-significant (RR = 1.02, 95% CI = 0.92–1.14, *p*-value = 0.68) across all studies (Supplementary Figure S9).

3. Overall Pooled Effect

**Figure 4:**
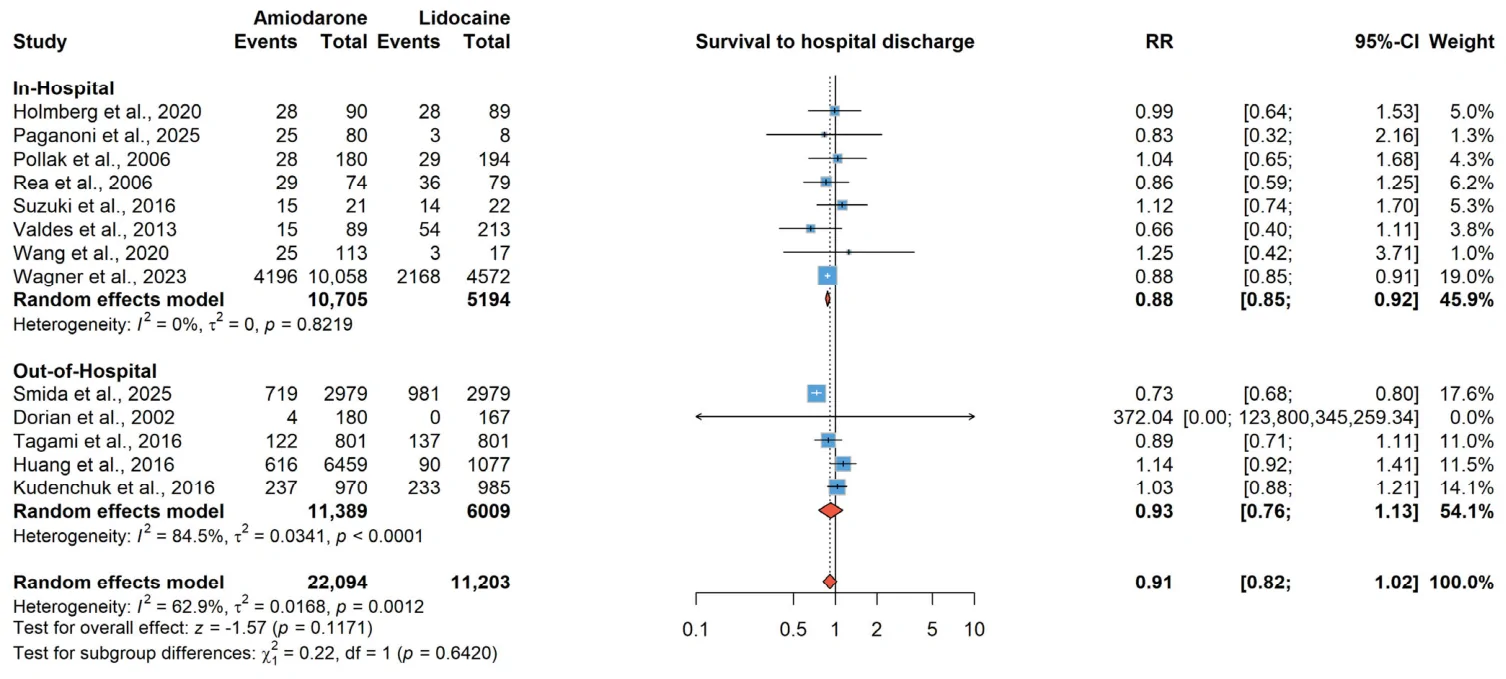
Forest Plot of Subgroup Analysis of Survival to Hospital Discharge [8,9,10,15,16,17,18,19,20,21,22,23,25]. Effect estimates compare amiodarone with lidocaine; RR < 1 favors lidocaine, whereas RR > 1 favors amiodarone. Squares indicate study-specific estimates, with their size reflecting study weight; horizontal lines represent 95% confidence intervals, and diamonds indicate pooled estimates.

When all studies (both IHCA and OHCA) were combined, the overall pooled risk ratio for survival to hospital discharge was 0.91 (95% CI = 0.82–1.02, *p* = 0.11), indicating no statistically significant survival benefit of using amiodarone over lidocaine in both in-hospital cardiac arrest and out-of-hospital cardiac arrest patients. Heterogeneity was moderate (I^2^ = 62.9%) (Figure 4).

##### 24 h Survival

**Figure 5:**
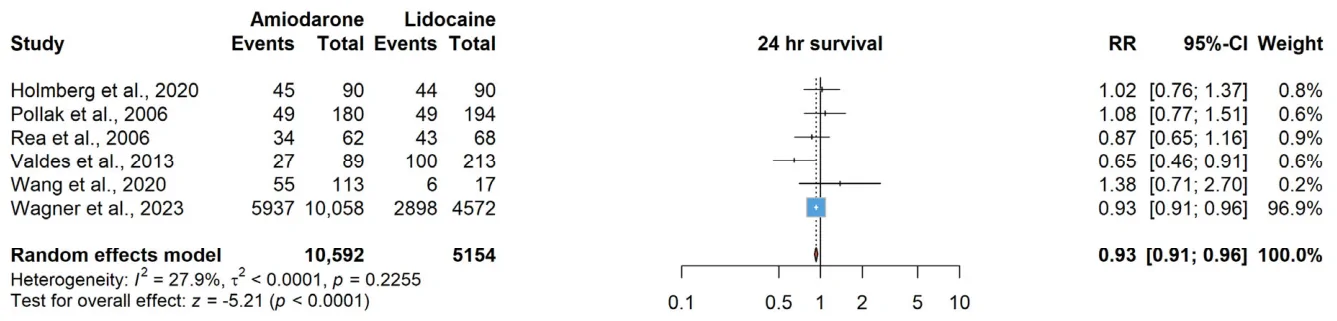
Forest Plot of 24 h Survival [10,15,16,18,19,21]. Effect estimates compare amiodarone with lidocaine; RR < 1 favors lidocaine, whereas RR > 1 favors amiodarone. Squares indicate study-specific estimates, with their size reflecting study weight; horizontal lines represent 95% confidence intervals, and diamonds indicate pooled estimates.

The effect of amiodarone versus lidocaine on survival in 24 h among patients experiencing in-hospital cardiac arrest was reported in a total of 6 included studies (*n* = 15,746). When collated and analyzed collectively, amiodarone, with an RR of 0.93, was associated with a moderate but significantly lower likelihood of 24 h survival compared to lidocaine (95% CI = 0.91–0.96, *p*-value < 0.0001). The overall heterogeneity was low to moderate (I^2^ = 28%), showing acceptable consistency among the included studies (Figure 5). To assess the robustness of the findings, leave-one-out sensitivity analysis was performed. These results suggested that no single study exerted a disproportionate influence on the overall effect size except Wagner et al., 2023 [10] (RR = 0.92, 95% CI = 0.75–1.13, *p*-value = 0.44) (Supplementary Figure S10).

##### Favourable Neurological Outcome

**Figure 6:**
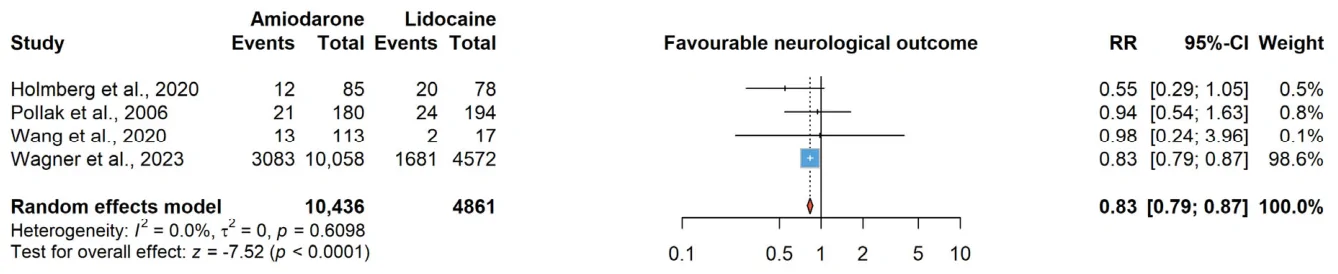
Forest Plot of Favourable Neurological Outcome [10,15,19,21]. Effect estimates compare amiodarone with lidocaine; RR < 1 favors lidocaine, whereas RR > 1 favors amiodarone. Squares indicate study-specific estimates, with their size reflecting study weight; horizontal lines represent 95% confidence intervals, and diamonds indicate pooled estimates.

Only four studies stated data on favorable neurological outcomes among patients treated with either amiodarone or lidocaine in the in-hospital cardiac arrest setting. The pooled random effects model showed that amiodarone use was associated with a 17% lower likelihood of achieving a favorable neurological outcome compared with lidocaine (RR = 0.83, 95% CI = 0.79–0.87, *p*-value < 0.0001). The heterogeneity across studies was negligible (I^2^ = 0%), indicating a high degree of consistency among included studies (Figure 6). Sensitivity analysis (leave-one-out method) was performed to evaluate the robustness of this aggregate estimate, but heterogeneity remained near zero across all iterations, along with uniform results, further confirming the stability of the findings (Supplementary Figure S11).

#### 3.4.2. Secondary Outcomes

##### Termination of Ventricular Fibrillation/Pulseless Ventricular Tachycardia

Three studies evaluated the likelihood of termination of shockable rhythms (ventricular fibrillation (VF) or pulseless ventricular tachycardia (VT)) by amiodarone in comparison with lidocaine following shock among in-hospital cardiac arrest patients. With a pooled risk ratio (RR) of 1.19, no significant difference between amiodarone and lidocaine was observed in achieving termination of VF/pulseless VT (95% CI = 0.24–5.95, *p*-value = 0.83). Considerable heterogeneity was observed across studies (I^2^ = 74%) (Supplementary Figure S12). Sensitivity analysis (leave-one-out method) was performed to assess the influence of individual studies on the combined estimate. The exclusion of Wang et al., 2020 [15], altered the pooled effect size towards statistical significance, with heterogeneity reduced to 0%, suggesting that the observed variability between studies was largely driven by it. (RR = 0.51, 95% CI = 0.30–0.89, *p*-value = 0.0167) (Supplementary Figure S13).

##### Duration of Cardiac Arrest (CA)

Two studies compared the duration of cardiac arrest (CA) among patients who received amiodarone versus lidocaine during in-hospital cardiac arrest. The pooled model showed that patients treated with amiodarone, when compared with those who received lidocaine, experienced a significantly longer duration of cardiac arrest by 7.46 min (MD = 7.46, 95% CI = 2.60–12.32, *p*-value = 0.002). There was no evidence of heterogeneity between studies (I^2^ = 0%), suggesting reliable findings across the included trials (Supplementary Figure S14).

##### Survival to ICU Admission (Out-of-Hospital Cardiac Arrest Subgroup)

Two studies assessed the effect of amiodarone versus lidocaine (*n* = 7433 vs. 2070) on survival to ICU admission following out-of-hospital cardiac arrest. The combined analysis of the two estimable trials failed to show any significant relation between survival-to-ICU admission and the two anti-arrhythmics (RR = 1.01, 95% CI = 0.93–1.11, *p*-value = 0.78). A moderate level of between-study variability was observed among the included studies (I^2^ = 34%) (Supplementary Figure S15).

### 3.5. Evidence from Randomized Controlled Trials

Randomized evidence was limited to two randomized controlled trials, Dorian et al. (2002) [8] and Kudenchuk et al. (2016) [9], both conducted in patients with out of hospital cardiac arrest (OHCA). The findings of these trials were reported separately for each outcome. For survival to hospital discharge, Dorian et al. (2002) [8] reported survival in 9/180 (5%) patients receiving amiodarone compared with 5/167 (3%) patients receiving lidocaine (RR = 1.67, 95% CI = 0.57–4.88; *p* = 0.34), indicating no statistically significant difference between treatments. Kudenchuk et al. (2016) [9], reporting return of spontaneous circulation (ROSC) at emergency department arrival, showed ROSC in 350/974 (35.9%) patients receiving amiodarone compared with 396/992 (39.9%) receiving lidocaine (RR = 0.90, 95% CI = 0.80–1.01), with no statistically significant difference between treatments; this was broadly consistent with the overall OHCA analysis, which included both randomized and observational studies (RR = 1.04). Survival to hospital discharge in the larger Kudenchuk et al. (2016) [9] trial occurred in 237/970 (24.4%) patients receiving amiodarone compared with 233/985 (23.7%) receiving lidocaine (RR = 1.03, 95% CI = 0.88–1.21), indicating no meaningful difference between treatments. The overall OHCA analysis, which included both randomized and observational studies, similarly showed no statistically significant difference in survival to hospital discharge (RR = 0.93, 95% CI = 0.76–1.13; *p* = 0.44). For survival to admission, Dorian et al. (2002) [8] reported survival to intensive care unit admission in 22.8% of patients receiving amiodarone compared with 12.0% of those receiving lidocaine (*p* = 0.009), favouring amiodarone. Kudenchuk et al. (2016) [9] reported survival to hospital admission in 45.7% of patients receiving amiodarone compared with 47.0% of those receiving lidocaine, with no significant difference between treatments. These findings were broadly consistent with the overall pooled analysis, which showed no statistically significant difference between amiodarone and lidocaine (RR = 1.01, 95% CI = 0.93–1.11; *p* = 0.78). Overall, the limited randomized evidence did not demonstrate a consistent superiority of amiodarone or lidocaine across the assessed outcomes. The limited and inconsistent randomized evidence supports cautious interpretation of the overall pooled estimate, which were informed predominantly by observational evidence.

### 3.6. Publication Bias

Assessment of potential small-study effects and funnel-plot asymmetry using Doi plots and the Luis Furuya-Kanamori (LFK) index demonstrated varying degrees of asymmetry across outcomes. For return of spontaneous circulation (ROSC), no significant asymmetry was observed in the in-hospital cardiac arrest (IHCA) subgroup (LFK = 0.45), suggesting little evidence of funnel-plot asymmetry (Supplementary Figure S16).

In contrast, regarding the survival to hospital discharge, major asymmetry was observed in both subgroups (LFK = 6.3 for OHCA; 2.35 for IHCA), indicating substantial funnel-plot asymmetry, that may reflect publication bias, small-study effects or methodological heterogeneity, therefore the results should be interpreted with caution (Supplementary Figures S17 and S18). However, asymmetry in doi plots is not specific to publication bias only and may also reflect substantial clinical and methodological heterogeneity among the included studies. In the present meta analysis, the differences in study design (randomized trials versus observational cohorts), sample size, patient populations, cardiac arrest characteristics, treatment protocols, and timing of anti-arrhythmic administration may have contributed to the observed asymmetry. Accordingly, the observed asymmetry cannot be attributed definitively to publication bias alone, and requires cautious interpretation of the pooled estimate for survival to hospital discharge.

The 24 h survival outcome demonstrated a minor asymmetry (LFK = 1.03), indicating minor funnel plot asymmetry with limited evidence of small-study effects on this assessment (Supplementary Figure S19).

For the remaining outcomes, including favorable neurological outcomes, termination of VF/pulseless VT, ROSC in the OHCA subgroup, survival to ICU admission, and duration of cardiac arrest, fewer than five studies were available. Thus, a formal assessment of publication bias was not performed as the LFK index is unreliable when only a small number of studies are included [27].

### 3.7. Adult Versus Pediatric Subgroup Analysis in In-Hospital Cardiac Arrest

A subgroup analysis was conducted for IHCA outcomes based on pediatric versus adult populations. Two studies, Holmberg et al., 2020 [21] and Valdes et al., 2014 [16], included pediatric populations, while the remaining six studies included adults. Subgroup analyses by age did not demonstrate significant differences between adult and pediatric populations across outcomes. In adults, amiodarone was associated with lower survival to hospital discharge (RR = 0.88, 95% CI: 0.85–0.92; I^2^ = 0%), lower 24 h survival (RR = 0.93, 95% CI: 0.91–0.96; I^2^ = 0%), and a lower likelihood of favorable neurological outcome (RR = 0.83, 95% CI: 0.80–0.88; I^2^ = 0%) compared with lidocaine. Pediatric estimates for these outcomes were non-significant and based on limited evidence. No significant adult–pediatric subgroup differences were observed for survival to hospital discharge (*p* = 0.764), 24 h survival (*p* = 0.579), or favorable neurological outcome (*p* = 0.208). ROSC was non-significant in both age groups, with no significant subgroup difference (*p* = 0.411) (Supplementary Figures S30–S33).

### 3.8. GRADE Certainty of Evidence Results

The GRADE assessment showed predominantly low to very low certainty of evidence across outcomes. In IHCA, certainty was very low for ROSC, survival to hospital discharge, and termination of VF/pVT, while 24 h survival, favorable neurological outcome, and cardiac arrest duration were rated as low certainty. In OHCA, certainty was very low for ROSC and survival to hospital discharge, whereas survival to ICU admission had moderate certainty. Certainty differed by the design of the contributing evidence: because both randomized trials enrolled out-of-hospital populations, all six IHCA outcomes were informed exclusively by observational cohorts, whereas the three OHCA outcomes drew on mixed randomized and observational evidence. Downgrading was driven principally by risk of bias, very serious for the exclusively observational IHCA outcomes and serious for the mixed OHCA outcomes, along with inconsistency, imprecision, and suspected publication bias for selected outcomes (Supplementary Table S3).

### 3.9. Bayesian Results

For the primary outcome of overall ROSC, Bayesian random-effects meta-analyses of 10 studies showed no clear difference between amiodarone and lidocaine (RR 0.96; 95% CrI, 0.86–1.11), with low-to-moderate heterogeneity (τ = 0.13; 95% CrI, 0.04–0.31) (Supplementary Figure S20). Similar findings were observed for in-hospital ROSC (6 studies; RR 0.97; 95% CrI, 0.82–1.11) and out-of-hospital ROSC (4 studies; RR 0.99; 95% CrI, 0.78–1.41) (Supplementary Figures S21 and S22). Posterior probabilities of benefit were modest for overall, in-hospital, and out-of-hospital ROSC (0.37, 0.36, and 0.45, respectively), indicating limited evidence that either treatment provided a clinically meaningful advantage. Results were robust to prior-sensitivity and leave-one-out analyses.

For 24 h survival, the pooled Bayesian RR was 0.93 (95% CrI, 0.79–1.09), while favorable neurological outcome yielded an RR of 0.82 (95% CrI, 0.60–1.06) (Supplementary Figures S23 and S24). Importantly, the credible intervals included the null for both outcomes, weakening the apparent benefit of lidocaine observed in the frequentist analysis. For in-hospital cardiac arrest duration, the pooled mean difference was 7.4 min (95% CrI, 2.5–12.3), supporting a longer arrest duration with amiodarone, although this estimate was sensitive to one influential study.

Among secondary outcomes, Bayesian analyses showed no clear treatment difference for termination of VF/pVT (RR, 0.70; 95% CrI, 0.37–1.54), out-of-hospital survival to ICU admission (RR, 1.01; 95% CrI, 0.77–1.33), or overall survival to hospital discharge (13 studies; RR, 0.92; 95% CrI, 0.81–1.05) (Supplementary Figures S25–S27). The results were similarly inconclusive for out-of-hospital survival to discharge (RR 0.93; 95% CrI, 0.74–1.23) and in-hospital survival to discharge (RR 0.89; 95% CrI, 0.79–1.05) (Supplementary Figures S28 and S29). Thus, Bayesian analyses largely corroborated the frequentist findings but did not support a clear superiority of either antiarrhythmic for most clinically important outcomes.

## 4. Discussion

We compared the effectiveness of amiodarone versus lidocaine across a wide spectrum of clinically meaningful outcomes in both IHCA and OHCA settings in this comprehensive meta-analysis of fourteen studies published over more than two decades. Our analysis included more than fifty thousand patients, making it one of the most extensive evaluations of these agents to date and the first to integrate both randomized and observational evidence, subgroup analyses, and Bayesian modeling. In doing so, we examined the long-standing hypothesis that amiodarone is inherently superior to lidocaine during resuscitation, a premise commonly adopted in clinical practice but not uniformly supported by empirical evidence. Overall, our findings demonstrated no significant difference between amiodarone and lidocaine in achieving ROSC, survival to hospital discharge, termination of ventricular fibrillation or pulseless ventricular tachycardia, or survival to ICU admission. Although lidocaine demonstrated statistically significant associations with 24 h survival and favorable neurological outcome in selected in-hospital analyses, these findings should be interpreted cautiously and should not be considered evidence of overall superiority. The marginal differences observed in favor of either agent did not translate into consistent or clinically meaningful superiority across the primary analyses, suggesting that the current evidence does not establish one antiarrhythmic as preferable to the other in most resuscitation scenarios. In addition, cardiac arrest duration was longer for patients treated with amiodarone, suggesting possible delays in rhythm termination or adverse hemodynamic effects. However, these associations may be influenced by substantial baseline differences and confounding by indication, particularly because most included studies were retrospective observational cohorts in which amiodarone may have been preferentially administered to patients with more refractory or prolonged arrests. Therefore, neither the absence nor the presence of statistically significant differences should be interpreted as definitive evidence of equivalence or superiority of either drug. Taken together, our findings suggest that the available evidence does not convincingly demonstrate clinically meaningful superiority of either amiodarone or lidocaine, and the observed short-term advantages associated with lidocaine should be regarded as hypothesis-generating rather than practice-defining. High-quality, carefully designed comparative studies are needed to isolate the pharmacological contribution of antiarrhythmic therapy from the numerous pre-treatment and post-resuscitation determinants of survival.

ROSC remains the cornerstone initial benchmark of resuscitation effectiveness, and our pooled analysis demonstrated no overall difference between amiodarone and lidocaine across ten studies that included both IHCA and OHCA settings. The considerable heterogeneity observed is multifactorial. First, the majority of included studies were observational and thus susceptible to confounding by indication, whereby patients with more severe physiologic instability or more refractory VF/pVT are more likely to receive amiodarone [8,9,17,18,21]. Typical resuscitation practice often favors amiodarone in more complex or prolonged cardiac arrests, which may bias outcomes toward poorer ROSC rates in amiodarone-treated patients. Importantly, the predominance of retrospective observational evidence means that these associations cannot be assumed to represent causal treatment effects. The two randomized trials provide a less confounded comparison, whereas the observational cohorts are more vulnerable to treatment-selection bias and differences in baseline arrest severity. Accordingly, the overall pooled estimate should be interpreted in the context of the different levels of evidence contributing to it. Second, there were variations in the timing of drug administration relative to defibrillation attempts, which is known to substantially influence ROSC probability. Earlier administration of anti-arrhythmics has been associated with higher ROSC rates [20], yet many observational cohorts did not report timing precisely. This limitation is particularly important because antiarrhythmic therapy is generally administered only after initial defibrillation attempts, meaning that differences in the interval between defibrillation and drug administration may substantially modify the apparent treatment effect. Third, differences in comorbidity prevalence—including hypotension, heart failure, ischemic cardiomyopathy, and chronic renal disease—may alter drug pharmacokinetics and pharmacodynamics, and these factors were not controlled uniformly across studies [10,15,20,25]. More broadly, clinically important determinants such as witnessed versus unwitnessed arrest, no-flow and low-flow duration, bystander CPR, CPR quality, timing and quality of defibrillation, initial myocardial substrate, and arrest etiology were inconsistently reported or controlled across the included studies. These factors may have a substantially greater influence on ROSC and subsequent survival than the choice of antiarrhythmic agent itself. Age-based subgroup analysis within the IHCA population did not demonstrate significant differences between adult and pediatric subgroups, although the pediatric evidence was limited.

In subgroup analysis, the OHCA cohort demonstrated a similar lack of statistical significance, although sensitivity analysis indicated that removal of the influential Dorian et al. ALIVE trial rendered the pooled effect significant [8]. This suggests that variability in study methodology, including differences in paramedic training, EMS protocols, and arrest etiology, strongly influences ROSC outcomes. The sensitivity analysis finding should therefore be interpreted as demonstrating the influence of an individual study rather than establishing a treatment advantage for either drug. Previous observational studies have compared amiodarone and lidocaine in patients with out-of-hospital cardiac arrest [22,23]. Nevertheless, combining IHCA and OHCA in an overall estimate should be interpreted cautiously because these populations differ substantially in arrest etiology, monitoring intensity, timing of intervention, EMS involvement, availability of immediate defibrillation, and post-resuscitation care. Although setting-specific subgroup analyses partially address this issue, they cannot completely eliminate the clinical and methodological heterogeneity introduced by pooling fundamentally different arrest environments. Although amiodarone and lidocaine differ in their electrophysiological and hemodynamic properties, including ion-channel effects and onset of action, it remains uncertain whether these pharmacological differences translate into meaningful differences in resuscitation outcomes [3,28,29,30,31]. The practical relevance of these pharmacological differences during cardiac arrest remains uncertain because drug administration occurs within a rapidly changing resuscitation environment in which defibrillation, CPR quality, perfusion, and arrest duration may dominate the probability of rhythm termination. ROSC is a highly time-sensitive marker, and small differences in the immediate resuscitation care could affect the outcomes substantially. Therefore, wide variation in resuscitation systems across studies could be considered a strong contributor to the observed heterogeneity.

Survival to hospital discharge, a more clinically meaningful outcome reflecting not only immediate resuscitation success but also post-resuscitation care, similarly showed no significant difference when aggregating all included studies. However, important subgroup differences emerged. In IHCA, lidocaine was associated with a modest but statistically significant improvement in survival to discharge. This association should be interpreted cautiously because the IHCA evidence is derived predominantly from observational studies and may reflect differences in patient selection, arrest characteristics, treatment indication, and post-resuscitation management rather than a direct pharmacological benefit of lidocaine. This finding aligns with several hospital-based observational studies suggesting that lidocaine may provide more stable short-term hemodynamics and avoid the hypotension often noted with amiodarone administration [10,21]. Hemodynamic instability immediately following ROSC is a major determinant of subsequent survival, and even transient vasodilatory episodes may precipitate myocardial stunning, impaired cerebral perfusion, and multi-organ dysfunction [32,33,34]. Lidocaine’s shorter half-life and more predictable pharmacokinetic profile may also contribute to improved survival in controlled in-hospital environments, where drug delivery is precise and continuous monitoring enables titration of post-resuscitation care [28,35]. Nevertheless, these mechanistic explanations should not be interpreted as confirmation of causality, particularly because the timing and intensity of post-resuscitation interventions may themselves differ between treatment groups.

In contrast, OHCA studies revealed substantial heterogeneity and no significant pooled effect, consistent with the prior literature showing that survival to discharge is strongly determined by pre-hospital factors such as bystander CPR, rapid defibrillation, and early airway management [36,37]. The contrast between IHCA and OHCA therefore further supports the importance of considering the entire chain of survival rather than interpreting antiarrhythmic selection as an isolated determinant of outcome. Our finding that removal of one influential study eliminated heterogeneity and produced consistent non-significant results suggests that methodological and population differences may have disproportionately influenced the pooled estimate. Moreover, the overall pooled estimate combining IHCA and OHCA failed to show a significant effect, reinforcing the notion that drug selection alone cannot overcome system-of-care variables that strongly dictate ultimate survival. Previous meta-analyses, largely limited to OHCA, similarly reported no significant survival-to-discharge benefit for either drug. Importantly, the available evidence should not be interpreted as demonstrating equivalence between the drugs; rather, the lack of a consistent difference indicates that the current evidence is insufficient to establish clinically meaningful superiority of either agent.

Our meta-analysis showed that lidocaine resulted in a significantly higher likelihood of 24 h survival than did amiodarone. Differences in the acute cardiovascular effects of amiodarone and lidocaine, together with variability in early post-resuscitation physiology and care, provide potential biological explanations for differences in short-term outcomes [35,38,39]; however, these mechanisms cannot establish causality. The 24 h survival finding should therefore be considered exploratory because most contributing evidence was observational and may be affected by residual confounding. Moreover, the frequentist association was not confirmed by the Bayesian analysis and became non-significant after the exclusion of Wagner et al. (2023) [10]. Accordingly, the observed improvement in early survival should not be interpreted as evidence that lidocaine provides a durable survival advantage, particularly in the absence of consistent benefit for later outcomes.

Favorable neurological outcome is arguably the most important metric of resuscitation success, as survival without meaningful recovery offers limited functional benefit. Only four IHCA studies evaluated this outcome, but the pooled analysis demonstrated a significantly higher likelihood of favorable neurological recovery with lidocaine. Given that only four IHCA studies contributed to this analysis and the broader evidence base is predominantly observational, this statistically significant finding should be regarded as preliminary rather than definitive evidence of neurological superiority. Neurological recovery after cardiac arrest is strongly influenced by cerebral perfusion, pre-arrest status, arrest duration, and post-resuscitation management, while the experimental and perioperative literature has proposed potential neurological effects of antiarrhythmic and anesthetic agents [40,41,42,43,44,45,46]. However, these mechanistic observations cannot establish a direct neuroprotective benefit of lidocaine in cardiac arrest. Our finding of zero heterogeneity strengthens confidence in the pooled estimate, and sensitivity analyses confirm robustness. Nevertheless, statistical consistency within a small number of studies does not eliminate the risk of systematic bias or establish causality. Prior meta-analyses focused primarily on OHCA and were unable to adequately examine neurological outcomes due to scarce data [47,48,49,50]. Our analysis provides novel evidence that lidocaine may hold clinically meaningful advantages regarding neurological preservation in in-hospital cardiac arrests. This finding should therefore be considered hypothesis-generating and warrants confirmation in adequately powered randomized studies rather than being interpreted as evidence that lidocaine is superior for neurological preservation.

The prior literature has shown conflicting results on defibrillation efficacy between the two agents, with some trials favoring amiodarone and others showing no difference [8,9]. Our findings support the notion that rhythm termination is highly dependent on timing, patient-specific factors, and system-level variations, rather than drug selection alone as we exhibit a high heterogeneity in our findings owing to many confounding factors. In particular, differences in the number and timing of defibrillation attempts before drug administration, the interval between defibrillation and antiarrhythmic administration, and the duration of refractory VF/pVT may materially influence the apparent efficacy of either agent.

Duration of cardiac arrest, assessed in two IHCA studies, was substantially longer in patients receiving amiodarone. Arrest duration may be strongly influenced by arrest severity, treatment sequence, and pharmacological differences between the two agents. Because amiodarone may have been preferentially administered in more refractory or prolonged arrests, the observed longer duration should primarily be interpreted as an association susceptible to confounding by indication rather than as a demonstrated drug effect, particularly because only two studies contributed to this outcome.

Among OHCA cases alone, survival to ICU admission did not differ significantly between amiodarone and lidocaine. The survival of OHCA seems to be determined significantly by other factors, mainly pre-hospital protocols and EMS resources, along with bystander CPR, rather than administration of medication. Similarly, other meta-analyses restricted to OHCA have not demonstrated a comparative early survival benefit [50]. Taken together, these findings reinforce that antiarrhythmic therapy represents only one component of a highly interdependent chain of survival. Future comparative studies should therefore aim to enroll more homogeneous populations and explicitly stratify or adjust for witnessed status, bystander CPR, no-flow and low-flow duration, initial rhythm and arrest etiology, timing of defibrillation, timing of drug administration relative to defibrillation, and standardized post-resuscitation care. In particular, shock-refractory VF/pVT should be analyzed separately where possible, and ischemic and non-ischemic etiologies should be distinguished because their underlying myocardial substrates and responses to therapy may differ. Without controlling these determinants, even very large studies and meta-analyses may continue to provide uncertain estimates of the isolated pharmacological effect of antiarrhythmic therapy. Finally, although publication bias was assessed using DOI plots and the LFK index, these methods have recognized limitations, particularly when the number of studies is small. Therefore, potential small-study effects and publication bias cannot be excluded for outcomes with limited available evidence, and such assessments should be interpreted as supportive rather than definitive. Overall, our findings support continued clinical uncertainty rather than a change in preferred antiarrhythmic therapy: the current evidence does not establish clinically meaningful superiority of amiodarone or lidocaine, and the apparent short-term advantages associated with lidocaine should be interpreted cautiously in light of the predominance of observational evidence, residual confounding, and substantial heterogeneity in the cardiac arrest populations and resuscitation systems studied.

## 5. Strengths

Assessment for publication bias showed variable asymmetry across outcomes. Using the LFK index and Doi plots, significant asymmetry was detected for survival to hospital discharge in both IHCA and OHCA subgroups, raising the possibility of small-study effects, selective publication, or methodological heterogeneity among smaller observational cohorts. Methodological evidence suggests that Doi plots and the LFK index may detect asymmetry more sensitively than conventional funnel-plot methods [51]. There was a minor asymmetry for 24 h survival, and ROSC in IHCA showed little asymmetry. Neurological outcome, IHCA ROSC, termination of VF/pVT, duration of arrest, and survival to ICU admission were supported by fewer than five studies in our analysis; thus, publication bias assessment was not reliably calculable, consistent with methodological recommendations against the evaluation of funnel plot asymmetry when too few studies are available for such an assessment. This limit represents a call for more robust and harmonized reporting for cardiac arrest trials to enable definitive conclusions in the future.

Thus, methodological comprehensiveness stands out as a distinguishing strength of our meta-analysis relative to the prior literature. Past meta-analyses have often confined themselves exclusively to OHCA or relied heavily on older RCT data, or they included only very few observational studies [47,48,49,50]. Our study combines trial and cohort evidence, stratified by IHCA and OHCA, with Bayesian modeling to provide an integrated and contemporary assessment of the effectiveness of anti-arrhythmics. The Bayesian approaches have advantages in employing prior probability distributions. They allow the evaluation of uncertainty in a more nuanced manner, particularly relevant in resuscitation research, given that sample sizes are often limited and outcome variability is large [26,52]. Our analysis constitutes the largest dataset comparing these agents. Extensive sensitivity analyses strengthen the robustness of this review further.

## 6. Bayesian Analysis Discussion

### 6.1. Rationale

We performed the Bayesian analysis as a complementary approach because the included evidence was clinically and methodologically heterogeneous and largely observational, making reliance on frequentist statistical significance alone potentially misleading. In addition to estimating pooled effects, the Bayesian framework allowed us to directly quantify the probability of a true treatment benefit and the uncertainty surrounding that effect, while testing whether the frequentist findings remained robust across alternative assumptions.

### 6.2. Message for Clinicians

Clinically, the Bayesian analyses did not demonstrate superiority of either amiodarone or lidocaine for most outcomes. The Bayesian results largely supported the frequentist conclusion that neither amiodarone nor lidocaine was clearly superior for ROSC, survival to hospital discharge, VF/pVT termination, or ICU admission. Importantly, Bayesian analysis weakened several apparent frequentist signals favoring lidocaine: the differences in 24 h survival, favorable neurological outcome, and IHCA survival to discharge were no longer clearly supported once uncertainty was incorporated. Clinically, posterior probabilities should be interpreted as the probability that one treatment truly provides benefit given the observed evidence and prespecified model assumptions, rather than as a conventional *p*-value. The generally modest probabilities of benefit and credible intervals spanning the null indicate considerable uncertainty and do not support a clinically meaningful advantage of either drug.

## 7. Limitations

Several limitations deserve consideration despite the strengths of our meta-analysis. There was a general lack of consistency in the reporting of baseline characteristics in the included studies. Many cohorts did not clearly detail the burden of comorbidities, timing of drug administration, number of defibrillation attempts, use of epinephrine, and quality of CPR, which strongly influences outcomes. In addition, eligibility was restricted to full-text articles published in English. Although no language filter was applied at the search stage, non-English-language reports identified during screening could not be reliably extracted and were excluded; relevant evidence published in other languages may therefore have been missed, and language bias cannot be excluded. Most of the included studies are observational, and these are intrinsically susceptible to confounding, selection bias, and survivorship bias. Patients who survive long enough to receive anti-arrhythmics may be systematically different from those who do not, and adjustment strategies vary widely. Our analysis included two RCTs; however, the limited number of trials and emphasis on OHCA limit the generalisability of randomized evidence, particularly in the in-hospital setting. The randomization quality was generally unclear and poor in some studies, and uniformity in drug dosages or delivery routes was not always maintained. Considerable heterogeneity was seen in several outcomes, largely because of the inclusion of heterogeneous populations, different study design and setting, variations in arrest characteristics, and differences in drug dosing or timing of administration. Sensitivity analyses revealed that multiple outcomes reached statistical significance after excluding various influential studies, reflecting the complexity of interpreting pooled estimates in the presence of methodological diversity. Publication bias assessment using LFK and Doi plots identified asymmetry for some outcomes, particularly survival to discharge, highlighting the importance of cautious interpretation. Sensitivity analyses indicated that some findings were influenced by individual studies; for example, the significant IHCA survival-to-discharge result became non-significant after exclusion of Wagner et al. (2023) [10]. Therefore, these findings should be interpreted cautiously, particularly where results were not robust to sensitivity analysis. Finally, while Bayesian modeling does strengthen inference, the quality of the input data remains a limiting factor. To improve precision and validity in future evidence, large-scale multicenter RCTs with standardized protocols for drug dosing, timing, and reporting are urgently needed.

## 8. Conclusions

In conclusion, our comprehensive meta-analysis integrating in-hospital and out-of-hospital cardiac arrest populations, randomized trials, observational cohorts, and Bayesian modeling did not demonstrate a consistent overall survival advantage of either amiodarone or lidocaine. In the in-hospital setting, lidocaine was associated with improved 24 h survival, favorable neurological recovery, and survival to hospital discharge in the frequentist analyses; however, these findings were not consistently confirmed by Bayesian analyses and should therefore be interpreted cautiously. Importantly, the limited randomized evidence, derived predominantly from OHCA populations, did not demonstrate clear superiority of either amiodarone or lidocaine. Across the overall pooled analyses, survival to hospital discharge, ROSC, and several other clinically relevant outcomes did not demonstrate a consistent difference between the two agents. Therefore, although selected in-hospital analyses suggested a potential association between lidocaine and more favorable short-term outcomes, these findings may be influenced by residual confounding and do not establish clinically meaningful superiority of either antiarrhythmic drug. These findings should be interpreted cautiously given the predominance of observational evidence, residual confounding, substantial clinical heterogeneity, and the sensitivity of some associations to the exclusion of influential studies. Further adequately powered randomized trials with standardized resuscitation and drug-administration protocols are needed to clarify whether meaningful differences exist between the two agents. 

## Figures and Tables

**Table 1:** Baseline characteristics of included studies.

Study ID	Country	Study Design	Hospital Setting	Intervention vs. Control	Sample Size (I vs. C)	Males (%)	Mean Age (SD)	Hypotension	Primary Outcome
Holmberg 2020 [21]	USA	Prospective cohort	In hospital	Amiodarone vs. Lidocaine	365 (148 vs. 217)	86 (58) vs. 115 (53)	NR	43 (29) vs. 66 (30)	ROSC
Paganoni 2025 [20]	USA	Retrospective, cohort	In hospital	Amiodarone vs. Lidocaine	88 (80 vs. 8)	NR	NR	NR	ROSC
Pollak 2006 [19]	Ontario	Retrospective, cohort	In hospital	Amiodarone vs. Lidocaine	374	NR	NR	NR	Survival of resuscitation
Rea 2006 [18]	USA	Retrospective, cohort	In hospital	Amiodarone vs. Lidocaine	153 (74 vs. 79)	49 (66) vs. 61 (77)	62 (14) vs. 64 (17)	3 (4) vs. 5 (6)	Survival at 24 h post cardiac arrest
Suzuki 2016 [17]	Japan	Mutlicentre, observational cohorts	In hospital	Amiodarone vs. Lidocaine	72 (21 vs. 22)	15 (71.48) vs. 15 (68.18)	67.3 (13.5) vs. 65.7 (11)	NR	drug switching
Valdes 2014 [16]	USA	Retrospective, cohort	In hospital	Amiodarone vs. Lidocaine	889 (171 vs. 295)	NR	NR	NR	ROSC
Wagner 2023 [10]	USA	Retrospective, cohort	In hospital	Amiodarone vs. Lidocaine	14,630 (10,058 vs. 4572)	2868 (62.7) vs. 6478 (64.4)	NR	950 (20.8) vs. 2224 (22.2)	ROSC
Wang 2020 [15]	Taiwan	Retrospective cohort	In hospital	Amiodarone vs. Lidocaine	130 (113 vs. 17)	76 (67.3) vs. 15 (88.2)	68.2 (14.9) vs. 59.1 (13.6)	30 (26.5) vs. 3 (17.6)	Termination of VF/pVT within 3 shocks
Dorian 2002 [8]	Canada	Double-blind RCT	Out hospital	Amiodarone vs. Lidocaine	347 (180 vs. 167)	NR	68 (14) vs. 66 (13)	NR	Survival to admission to the ICU
Huang 2017 [25]	Taiwan	Retrospective cohort	Out hospital	Amiodarone vs. Lidocaine	7536 (6459 vs. 1077)	4489 (69.50) vs. 686 (63.70)	64.75 (16.18) vs. 66.83 (16.28)	NR	1-year survival
Kishihara 2022 [24]	Japan	Retrospective cohort	Out hospital	Amiodarone vs. Lidocaine	252 (189 vs. 63)	143 (75.7) vs. 49 (77.8)	69 (13.44) vs. 69.05 (15.14)	NR	30d survival
Kudenchuk 2016 [9]	North America	Double Blind, RCT	Out hospital	Amiodarone vs. Lidocaine	1967 (974 vs. 993)	762 (78.3) vs. 816 (82.2)	63.7 (14) vs. 63 (14.7)	NR	Survival to hospital discharge
Smida 2025 [23]	USA	Restrospective cohort	Out hospital	Amiodarone vs. Lidocaine	23,263 (20,284 vs. 2979)	NR	61.7 (12.6) vs. 62 (12.6)	NR	ROSC
Tagami 2016 [22]	Japan	Retrospective cohort	Out hospital	Amiodarone vs. Lidocaine	1602 (801 vs. 801)	600 (74.9) vs. 616 (76.9)	66.9 (14.3) vs. 67.3 (14.8)	NR	Survival to hospital discharge

**Abbreviations:** RCT: Randomized Controlled Trial; NR: Not Reported; USA: United States of America; ROSC: Return of Spontaneous Circulation; VF: Ventricular Fibrillation; pVT: Pulseless Ventricular Tachycardia.

**Table 2:** Outcome table of included studies.

Outcome/Setting	Studies, *n*	Participants A/L	Primary Effect (95% CI)	*p*-Value	I^2^	Direction of Benefit	Sensitivity Analysis: Study Omitted & Interpretation	Effect After SA (95% CI)	*p*-Value After SA	I^2^ After SA
ROSC—Overall	10	16,605/9334	RR 0.96 (0.87–1.06)	0.4028	75.1%	Numerically favors lidocaine (NS)	No influential study identified; pooled inference remained unchanged.	-		
ROSC—OHCA	4	5995/4240	RR 1.04 (0.78–1.38)	0.7832	77.0%	Numerically favors amiodarone (NS)	Dorian et al., 2002 [8]; omission changed statistical inference and reduced heterogeneity.	RR 0.88 (0.81–0.95)	0.0017	41.8%
ROSC—IHCA	6	10,610/5094	RR 0.97 (0.87–1.07)	0.5193	44.0%	Numerically favors lidocaine (NS)	No omission changed inference. Valdes et al., 2014 [16] produced the largest heterogeneity reduction.	RR 0.99 (0.97–1.01)	0.4288	0%
Survival to hospital discharge—Overall	13	22,094/11,203	RR 0.91 (0.82–1.02)	0.1171	62.9%	Numerically favors lidocaine (NS)	No influential study identified; pooled inference remained unchanged.	-		
Survival to hospital discharge—IHCA	8	10,705/5194	RR 0.88 (0.85–0.92)	<0.0001	0%	**Favors lidocaine**	Wagner et al., 2023 [10]; omission removed statistical significance.	RR 0.94 (0.78–1.13)	0.4965	0%
Survival to hospital discharge—OHCA	5	11,389/6009	RR 0.93 (0.76–1.13)	0.4498	84.5%	Numerically favors lidocaine (NS)	Smida et al., 2025 [23]; omission eliminated heterogeneity while inference remained non-significant.	RR 1.02 (0.92–1.14)	0.6820	0%
24 h survival—IHCA	6	10,592/5154	RR 0.93 (0.91–0.96)	<0.0001	27.9%	**Favors lidocaine**	Wagner et al., 2023 [10]; omission removed statistical significance.	RR 0.92 (0.75–1.13)	0.4418	42.0%
Favorable neurological outcome—IHCA	4	10,436/4861	RR 0.83 (0.79–0.87)	<0.0001	0%	**Favors lidocaine**	Wagner et al., 2023 [10]; omission removed statistical significance.	RR 0.77 (0.49–1.19)	0.2339	0%
Termination of VF/pVT—IHCA	3	208/118	RR 1.19 (0.24–5.95)	0.8357	73.5%	Numerically favors amiodarone (NS)	Wang et al., 2020 [15]; omission changed inference to favor lidocaine and eliminated heterogeneity.	RR 0.51 (0.30–0.89)	0.0167	0%
Survival to ICU admission—OHCA	2	7433/2070	RR 1.01 (0.93–1.11)	0.7846	34.0%	Numerically favors amiodarone (NS)	Leave-one-out pooled sensitivity analysis was not informative because exclusion of either study leaves a single-study estimate.	-		
Duration of cardiac arrest—IHCA	2	215/235	MD +7.46 min (2.60–12.32)	0.0026	0%	**Favors lidocaine (shorter arrest duration)**	Leave-one-out pooled sensitivity analysis was not informative because exclusion of either study leaves a single-study estimate.	-		

**Notes:** Effect estimates compare amiodarone (A) with lidocaine (L). For RR-based outcomes, RR < 1 favors lidocaine and RR > 1 favors amiodarone. For cardiac-arrest duration, a positive MD indicates a longer duration with amiodarone. SA denotes leave-one-out sensitivity analysis. Where an influential omission was identified, the table reports the omitted study together with the recalculated effect estimate, *p*-value, and I^2^. A dash indicates that no single influential leave-one-out result was reported or that an informative leave-one-out analysis was not presented. **Abbreviations:** A, amiodarone; L, lidocaine; CI, confidence interval; IHCA, in-hospital cardiac arrest; OHCA, out-of-hospital cardiac arrest; ICU, intensive care unit; MD, mean difference; pVT, pulseless ventricular tachycardia; ROSC, return of spontaneous circulation; RR, risk ratio; SA, sensitivity analysis; VF, ventricular fibrillation, *n*, number of studies, LOO, leave-one-out.

## Data Availability

All data generated or analyzed during this study are included in this published article and its Supplementary Materials.

## Supplementary Materials

The following supporting information can be downloaded at https://www.mdpi.com/article/10.3390/jcm15187274/s1, Table S1: PRISMA 2020 Checklist; Table S2: Search Strategy Table; Table S3: GRADE Assessment of the Certainty of Evidence for Amiodarone versus Lidocaine in Patients with Shockable Cardiac Arrest; Table S4: Study-Level Reporting of Potential Sources of Clinical Heterogeneity; Figure S1: Quality Assessment of Included Randomized Controlled Trials Using the Cochrane Risk of Bias 2 Tool; Figure S2: Quality Assessment of Included Observational Studies Using the ROBINS-I Tool; Figure S3: Forest Plot of Return of Spontaneous Circulation in Out-of-Hospital Cardiac Arrest; Figure S4: Leave-One-Out Plot of Return of Spontaneous Circulation in Out-of-Hospital Cardiac Arrest; Figure S5: Forest Plot of Return of Spontaneous Circulation in In-Hospital Cardiac Arrest; Figure S6: Leave-One-Out Plot of Return of Spontaneous Circulation in In-Hospital Cardiac Arrest; Figure S7: Leave-One-Out Plot of Survival to Hospital Discharge in In-Hospital Cardiac Arrest; Figure S8: Forest Plot of Survival to Hospital Discharge in Out-of-Hospital Cardiac Arrest; Figure S9: Leave-One-Out Plot of Survival to Hospital Discharge in Out-of-Hospital Cardiac Arrest; Figure S10: Leave-One-Out Plot of 24-h Survival; Figure S11: Leave-One-Out Plot of Favourable Neurological Outcome; Figure S12: Forest Plot of Termination of Ventricular Fibrillation/Pulseless Ventricular Tachycardia; Figure S13: Leave-One-Out Plot of Termination of Ventricular Fibrillation/Pulseless Ventricular Tachycardia; Figure S14: Forest Plot of Duration of Cardiac Arrest; Figure S15: Forest Plot of Survival to Intensive Care Unit Admission; Figure S16: LFK Plot of Return of Spontaneous Circulation in In-Hospital Cardiac Arrest; Figure S17: LFK Plot of Survival to Hospital Discharge in In-Hospital Cardiac Arrest; Figure S18: LFK Plot of Survival to Hospital Discharge in Out-of-Hospital Cardiac Arrest; Figure S19: LFK Plot of 24-h Survival; Figure S20: Bayesian Forest Plot for Overall Return of Spontaneous Circulation; Figure S21: Bayesian Forest Plot for the In-Hospital Cardiac Arrest Return of Spontaneous Circulation Subgroup; Figure S22: Bayesian Forest Plot for the Out-of-Hospital Cardiac Arrest Return of Spontaneous Circulation Subgroup; Figure S23: Bayesian Forest Plot for 24-h In-Hospital Survival; Figure S24: Bayesian Forest Plot for In-Hospital Favourable Neurological Outcome; Figure S25: Bayesian Forest Plot for In-Hospital Termination of Ventricular Fibrillation/Pulseless Ventricular Tachycardia with Shock; Figure S26: Bayesian Forest Plot for Survival to Intensive Care Unit Admission Following Out-of-Hospital Cardiac Arrest; Figure S27: Bayesian Forest Plot for Overall Survival to Hospital Discharge; Figure S28: Bayesian Forest Plot for the Out-of-Hospital Survival-to-Hospital-Discharge Subgroup; Figure S29: Bayesian Forest Plot for the In-Hospital Survival-to-Hospital-Discharge Subgroup; Figure S30: Forest Plot of Adult versus Pediatric Subgroup Analysis for In-Hospital Survival to Hospital Discharge; Figure S31: Forest Plot of Adult versus Pediatric Subgroup Analysis for In-Hospital 24-h Survival; Figure S32: Forest Plot of Adult versus Pediatric Subgroup Analysis for In-Hospital Favourable Neurological Outcome; Figure S33: Forest Plot of Adult versus Pediatric Subgroup Analysis for Return of Spontaneous Circulation in In-Hospital Cardiac Arrest.
