# Supplementary material for: Comparative Effectiveness of Amiodarone Versus Lidocaine as Antiarrhythmic Therapy for Cardiac Arrest with Shockable Rhythms: A Systematic Review and Meta-Analysis
Source: J Clin Med. 2026 Sep 19;15(18):7274. doi: 10.3390/jcm15187274 (PMC13607829; doi:10.3390/jcm15187274)
Supplement: Supplementary file 1 [file jcm-15-07274-s001.zip › jcm-4462995-supplementary.pdf]

Section and Topic	Item #	Checklist item	Location where item is reported
**TITLE**			
Title	1	Identify the report as a systematic review.	Page 1
**ABSTRACT**			
Abstract	2	See the PRISMA 2020 for Abstracts checklist.	Page 1-2
**INTRODUCTION**			
Rationale	3	Describe the rationale for the review in the context of existing knowledge.	Pages 2–3
Objectives	4	Provide an explicit statement of the objective(s) or question(s) the review addresses.	Page 3, final paragraph of the Introduction
**METHODS**			
Eligibility criteria	5	Specify the inclusion and exclusion criteria for the review and how studies were grouped for the syntheses.	Pages 3–4, Section 2.3
Information sources	6	Specify all databases, registers, websites, organisations, reference lists and other sources searched or consulted to identify studies. Specify the date when each source was last searched or consulted.	Page 3, Section 2.2
Search strategy	7	Present the full search strategies for all databases, registers and websites, including any filters and limits used.	Page 3, Section 2.2; Supplementary File Pages 1–2, Supplementary Table S1
Selection process	8	Specify the methods used to decide whether a study met the inclusion criteria of the review, including how many reviewers screened each record and each report retrieved, whether they worked independently, and if applicable, details of automation tools used in the process.	Page 4, Section 2.3
Data collection process	9	Specify the methods used to collect data from reports, including how many reviewers collected data from each report, whether they worked independently, any processes for obtaining or confirming data from study investigators, and if applicable, details of automation tools used in the process.	Page 4, Section 2.4
Data items	10a	List and define all outcomes for which data were sought. Specify whether all results that were compatible with each outcome domain in each study were sought (e.g. for all measures, time points, analyses), and if not, the methods used to decide which results to collect.	Page 4, Section 2.5
	10b	List and define all other variables for which data were sought (e.g. participant and intervention characteristics, funding sources). Describe any assumptions made about any missing or unclear information.	Page 4, Section 2.4; Pages 6–7, Section 3.2 and Table 1
Study risk of bias assessment	11	Specify the methods used to assess risk of bias in the included studies, including details of the tool(s) used, how many reviewers assessed each study and whether they worked independently, and if applicable, details of automation tools used in	Page 4, Section 2.6; Pages 7–8, Section 3.3; Supplementary File Pages 3–4, Figures S1–S2

Section and Topic	Item #	Checklist item	Location where item is reported
		the process.	
Effect measures	12	Specify for each outcome the effect measure(s) (e.g. risk ratio, mean difference) used in the synthesis or presentation of results.	Pages 4–5, Sections 2.7–2.8
Synthesis methods	13a	Describe the processes used to decide which studies were eligible for each synthesis (e.g. tabulating the study intervention characteristics and comparing against the planned groups for each synthesis (item #5)).	Pages 3–5, Sections 2.3, 2.5 and 2.7
	13b	Describe any methods required to prepare the data for presentation or synthesis, such as handling of missing summary statistics, or data conversions.	Page 5, Section 2.8
	13c	Describe any methods used to tabulate or visually display results of individual studies and syntheses.	Pages 4–5, Section 2.7; Pages 8–12, Table 2 and Figures 2–6; Supplementary File Pages 5–17, Figures S3–S29
	13d	Describe any methods used to synthesize results and provide a rationale for the choice(s). If meta-analysis was performed, describe the model(s), method(s) to identify the presence and extent of statistical heterogeneity, and software package(s) used.	Pages 4–5, Sections 2.7–2.8
	13e	Describe any methods used to explore possible causes of heterogeneity among study results (e.g. subgroup analysis, meta-regression).	Page 5, Section 2.7
	13f	Describe any sensitivity analyses conducted to assess robustness of the synthesized results.	Page 5, Sections 2.7–2.8; Pages 9–14, Sections 3.4–3.6; Supplementary Figures S4, S6, S7, S9, S10, S11 and S13
Reporting bias assessment	14	Describe any methods used to assess risk of bias due to missing results in a synthesis (arising from reporting biases).	Page 4, Section 2.6; Page 12, Section 3.5; Supplementary File Pages 9–12, Figures S16–S19
Certainty assessment	15	Describe any methods used to assess certainty (or confidence) in the body of evidence for an outcome.	No formal certainty-of-evidence assessment was performed. Bayesian analyses were used as complementary robustness analyses (Page 5, Section 2.8)
**RESULTS**			
Study selection	16a	Describe the results of the search and selection process, from the number of records identified in the search to the number of studies included in the review, ideally using a flow diagram.	Pages 5–6, Section 3.1 and Figure 1
	16b	Cite studies that might appear to meet the inclusion criteria, but which were excluded, and explain why they were excluded.	Page 6, Figure 1 (Partial:we showed reasons, but excluded studies are not individually cited)
Study characteristics	17	Cite each included study and present its characteristics.	Pages 6–7, Section 3.2 and Table 1

Section and Topic	Item #	Checklist item	Location where item is reported
Risk of bias in studies	18	Present assessments of risk of bias for each included study.	Pages 7–8, Section 3.3; Supplementary File Pages 3–4, Figures S1–S2
Results of individual studies	19	For all outcomes, present, for each study: (a) summary statistics for each group (where appropriate) and (b) an effect estimate and its precision (e.g. confidence/credible interval), ideally using structured tables or plots.	Pages 8–14, Sections 3.4–3.6, Table 2 and Figures 2–6; Supplementary File Pages 5–17, Figures S3–S29
Results of syntheses	20a	For each synthesis, briefly summarise the characteristics and risk of bias among contributing studies.	Pages 6–8, Sections 3.2–3.3 and Table 1; Supplementary Figures S1–S2
	20b	Present results of all statistical syntheses conducted. If meta-analysis was done, present for each the summary estimate and its precision (e.g. confidence/credible interval) and measures of statistical heterogeneity. If comparing groups, describe the direction of the effect.	Pages 8–14, Sections 3.4–3.6, Table 2 and Figures 2–6; Supplementary Figures S3–S15 and S20–S29
	20c	Present results of all investigations of possible causes of heterogeneity among study results.	Pages 9–12, Sections 3.4.1–3.4.2; Pages 14–19, Discussion and Limitations
	20d	Present results of all sensitivity analyses conducted to assess the robustness of the synthesized results.	Pages 9–14, Sections 3.4–3.6; Supplementary Figures S4, S6, S7, S9, S10, S11 and S13
Reporting biases	21	Present assessments of risk of bias due to missing results (arising from reporting biases) for each synthesis assessed.	Page 12, Section 3.5; Supplementary File Pages 9–12, Figures S16–S19
Certainty of evidence	22	Present assessments of certainty (or confidence) in the body of evidence for each outcome assessed.	Not applicable (no formal certainty-of-evidence assessment was performed)
**DISCUSSION**			
Discussion	23a	Provide a general interpretation of the results in the context of other evidence.	Pages 14–18, Discussion and Bayesian Approach
	23b	Discuss any limitations of the evidence included in the review.	Pages 18–19, Limitations
	23c	Discuss any limitations of the review processes used.	Pages 18–19, Limitations
	23d	Discuss implications of the results for practice, policy, and future research.	Pages 18–19, Limitations and Conclusion
**OTHER INFORMATION**			
Registration and protocol	24a	Provide registration information for the review, including register name and registration number, or state that the review was not registered.	Page 3, Section 2.1; PROSPERO CRD420251276136
	24b	Indicate where the review protocol can be accessed, or state that a protocol was not prepared.	Page 3, Section 2.1; PROSPERO CRD420251276136
	24c	Describe and explain any amendments to information provided at registration or in the protocol.	No amendments were made to the eligibility

Section and Topic	Item #	Checklist item	Location where item is reported
			criteria, outcomes, or analytical methods after protocol registration
Support	25	Describe sources of financial or non-financial support for the review, and the role of the funders or sponsors in the review.	Page 20, Funding
Competing interests	26	Declare any competing interests of review authors.	Page 20, Conflict of Interest Disclosure
Availability of data, code and other materials	27	Report which of the following are publicly available and where they can be found: template data collection forms; data extracted from included studies; data used for all analyses; analytic code; any other materials used in the review.	Page 20, Data Availability Statement

From: Page MJ, McKenzie JE, Bossuyt PM, Boutron I, Hoffmann TC, Mulrow CD, et al. The PRISMA 2020 statement: an updated guideline for reporting systematic reviews. BMJ 2021;372:n71. doi: 10.1136/bmj.n71. This work is licensed under CC BY 4.0. To view a copy of this license, visit https://creativecommons.org/licenses/by/4.0/

Supplementary Table S1: PRISMA 2020 Checklist [

Database	Search Strategy	Results
PUBMED	("Heart Arrest"[Mesh] OR "Out-of-Hospital Cardiac Arrest"[Mesh] OR "Death, Sudden, Cardiac"[Mesh] OR cardiac arrest[tiab] OR sudden cardiac arrest[tiab] OR cardiopulmonary arrest[tiab] OR CPR[tiab] OR "Ventricular Fibrillation"[Mesh] OR ventricular fibr[tiab] OR "Tachycardia, Ventricular"[Mesh] OR ventricular tachy[tiab] OR VF[tiab] OR VT[tiab] OR pulseless ventricular tachycardia[tiab] OR shockable rhythm*[tiab] OR VF arrest[tiab] OR VT arrest[tiab] OR pulseless arrhythmia[tiab]) AND ("in-hospital"[tiab] OR IHCA OR "in-hospital cardiac arrest"[tiab] OR "hospital cardiac arrest"[tiab] OR "out-of-hospital"[tiab] OR "out of hospital"[tiab] OR OHCA OR prehospital[tiab] OR pre-hospital[tiab] OR "Emergency Medical Services"[Mesh] OR EMS[tiab]) AND ("Amiodarone"[Mesh] OR "Amiodarone Hydrochloride"[Supplementary Concept] OR amiodarone[tiab] OR Cordarone[tiab] OR Pacerone[tiab] OR Nexterone[tiab] OR "class III antiarrhythmic"[tiab] OR "class III anti-arrhythmic"[tiab] OR "Lidocaine"[Mesh] OR "lidocaine hydrochloride"[Supplementary Concept] OR lidocaine[tiab] OR lignocaine[tiab] OR Xylocaine[tiab] OR "class IB antiarrhythmic"[tiab] OR "class IB anti-arrhythmic"[tiab])	342
ScienceDirect	("cardiac arrest" OR "ventricular fibrillation" OR "ventricular tachycardia") AND (IHCA OR OHCA OR prehospital OR EMS) AND (amiodarone) AND (lidocaine)	439
Embase	('cardiac arrest'/exp OR 'sudden cardiac arrest' OR 'cardiopulmonary arrest' OR CPR OR 'ventricular fibrillation'/exp OR 'ventricular fibrillation' OR VF OR 'ventricular tachycardia'/exp OR 'ventricular tachycardia' OR VT OR 'pulseless ventricular tachycardia' OR 'shockable rhythm' OR 'shockable rhythms' OR 'shockable cardiac arrest' OR 'shockable cardiac rhythms' OR 'VF arrest' OR 'VT arrest' OR 'pulseless arrhythmia') AND ('in hospital' OR IHCA OR 'in-hospital cardiac arrest' OR 'hospital cardiac arrest' OR 'out of hospital' OR 'out-of-hospital cardiac arrest' OR OHCA OR prehospital OR 'pre-hospital' OR 'emergency health service'/exp	112

	OR EMS) AND ('amiodarone'/exp OR amiodarone OR 'amiodarone hydrochloride' OR Cordarone OR Pacerone OR Nexterone OR 'class III anti-arrhythmic' OR 'class III antiarrhythmic') AND ('lidocaine'/exp OR lidocaine OR lignocaine OR xylocaine OR 'lidocaine hydrochloride' OR 'class IB anti-arrhythmic' OR 'class IB antiarrhythmic')	
COCHRANE	#1 MeSH descriptor: [Heart Arrest] explode all trees<br>#2 (Heart Arrest):ti,ab,kw<br>#3 MeSH descriptor: [Out-of-Hospital Cardiac Arrest] explode all trees<br>#4 (Out-of-Hospital Cardiac Arrest):ti,ab,kw<br>#5 MeSH descriptor: [Death, Sudden, Cardiac] explode all trees<br>#6 (Death, Sudden, Cardiac):ti,ab,kw<br>#7 (cardiac arrest):ti,ab,kw<br>#8 (sudden cardiac arrest):ti,ab,kw<br>#9 (cardiopulmonary arrest):ti,ab,kw<br>#10 (CPR):ti,ab,kw<br>#11 (Ventricular Fibrillation):ti,ab,kw<br>#12 MeSH descriptor: [Tachycardia, Ventricular] explode all trees<br>#13 (Tachycardia, Ventricular):ti,ab,kw<br>#14 (ventricular tachy):ti,ab,kw<br>#15 (pulseless ventricular tachycardia):ti,ab,kw<br>#16 (shockable rhythm):ti,ab,kw<br>#17 (shockable rhythms):ti,ab,kw<br>#18 (shockable cardiac arrest):ti,ab,kw<br>#19 (shockable cardiac rhythms):ti,ab,kw<br>#20 (VF arrest):ti,ab,kw<br>#21 (VT arrest):ti,ab,kw<br>#22 (pulseless arrhythmia):ti,ab,kw<br>#23 #1 OR #2 OR #3 OR #4 OR #5 OR #6 OR #7 OR #8 OR #9 OR #10 OR #11 OR #12 OR #13 OR #14 OR #15 OR #16 OR #17 OR #18 OR #19 OR #20 OR #21 OR #22<br>#24 (in-hospital cardiac arrest):ti,ab,kw<br>#25 (hospital cardiac arrest):ti,ab,kw	37

	#26 (IHCA):ti,ab,kw<br>#27 (out-of-hospital):ti,ab,kw<br>#28 (out of hospital):ti,ab,kw<br>#29 (OHCA):ti,ab,kw<br>#30 MeSH descriptor: [Emergency Medical Services] explode all trees<br>#31 (EMS):ti,ab,kw<br>#32 #24 OR #25 OR #26 OR #27 OR #28 OR #29 OR #30 OR #31<br>#33 MeSH descriptor: [Amiodarone] explode all trees<br>#34 (Amiodarone):ti,ab,kw<br>#35 (Amiodarone Hydrochloride):ti,ab,kw<br>#36 (Cordarone):ti,ab,kw<br>#37 (class III antiarrhythmic):ti,ab,kw<br>#38 (class III anti-arrhythmic):ti,ab,kw<br>#39 #33 OR #34 OR #35 OR #36 OR #37 OR #38<br>#40 MeSH descriptor: [Lidocaine] explode all trees<br>#41 (lidocaine hydrochloride):ti,ab,kw<br>#42 (lidocaine):ti,ab,kw<br>#43 (lignocaine):ti,ab,kw<br>#44 (Xylocaine):ti,ab,kw<br>#45 (class IB antiarrhythmic):ti,ab,kw<br>#46 #40 OR #41 OR #42 OR #43 OR #44 OR #45<br>#47 #23 AND #32 AND #39 AND #46	

Supplementary Table S2: Search Strategy Table

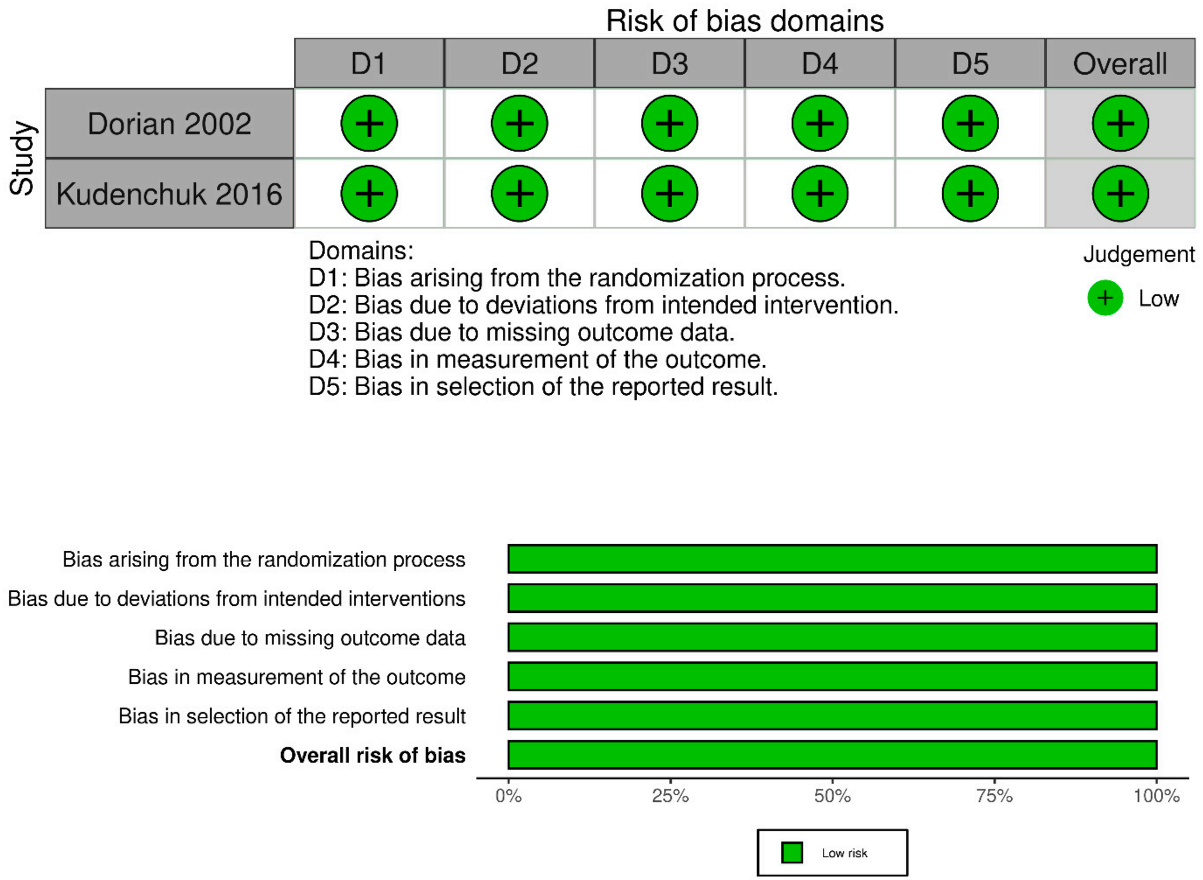

Supplementary Figure S1: Quality Assessment of Included Randomized Controlled Trials Using the Cochrane Risk of Bias 2 Tool

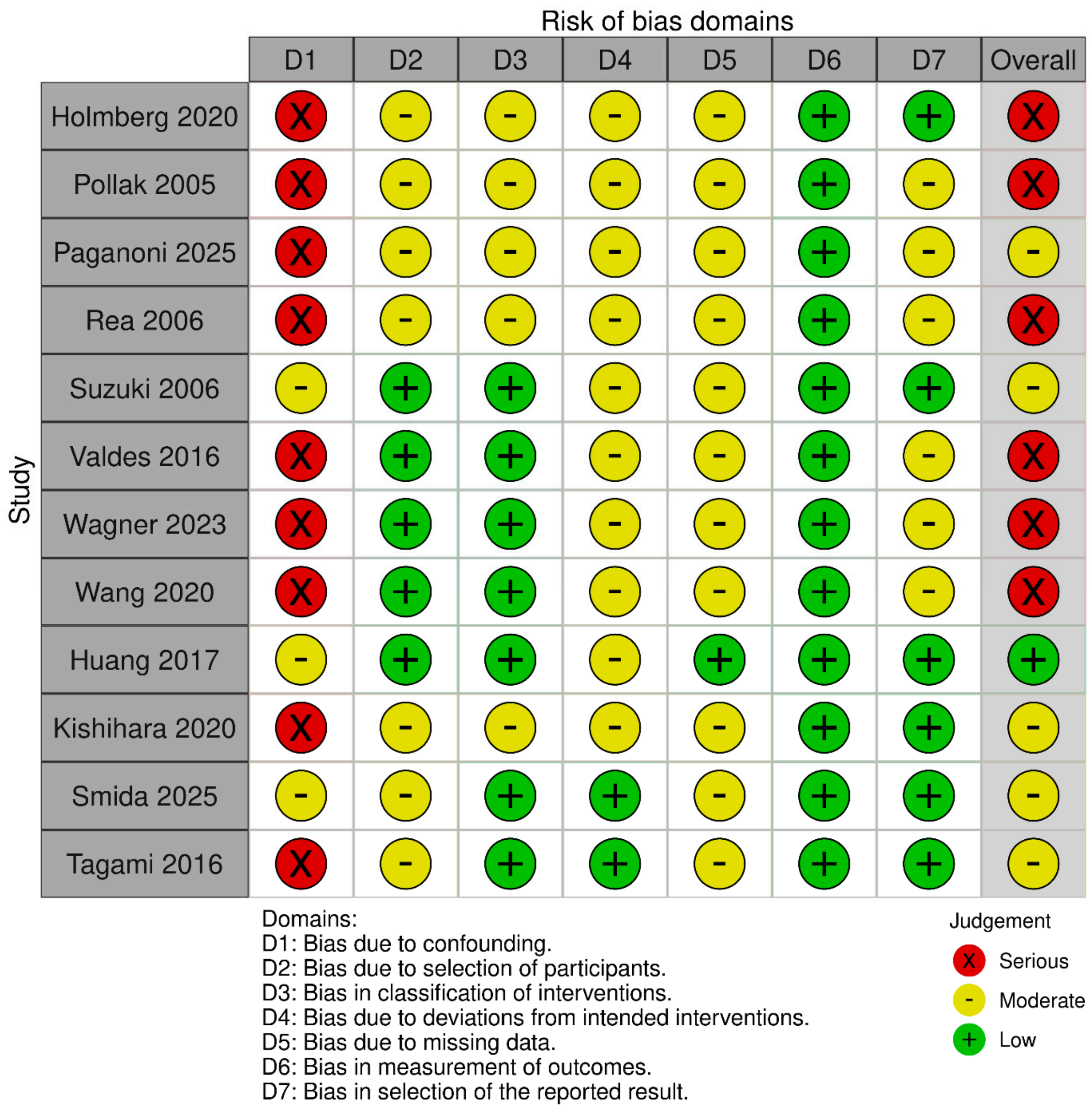
Risk of bias domains
D1 D2 D3 D4 D5 D6 D7 Overall
Study
Holmberg 2020 X - - - - + + X
Pollak 2005 X - - - - + - X
Paganoni 2025 X - - - - + - -
Rea 2006 X - - - - + - X
Suzuki 2006 - + + - - + + -
Valdes 2016 X + + - - + - X
Wagner 2023 X + + - - + - X
Wang 2020 X + + - - + - X
Huang 2017 - + + - + + + +
Kishihara 2020 X - - - - + + -
Smida 2025 - - + + - + + -
Tagami 2016 X - + + - + + -
Domains:
D1: Bias due to confounding.
D2: Bias due to selection of participants.
D3: Bias in classification of interventions.
D4: Bias due to deviations from intended interventions.
D5: Bias due to missing data.
D6: Bias in measurement of outcomes.
D7: Bias in selection of the reported result.
Judgement
X Serious
- Moderate
+ Low

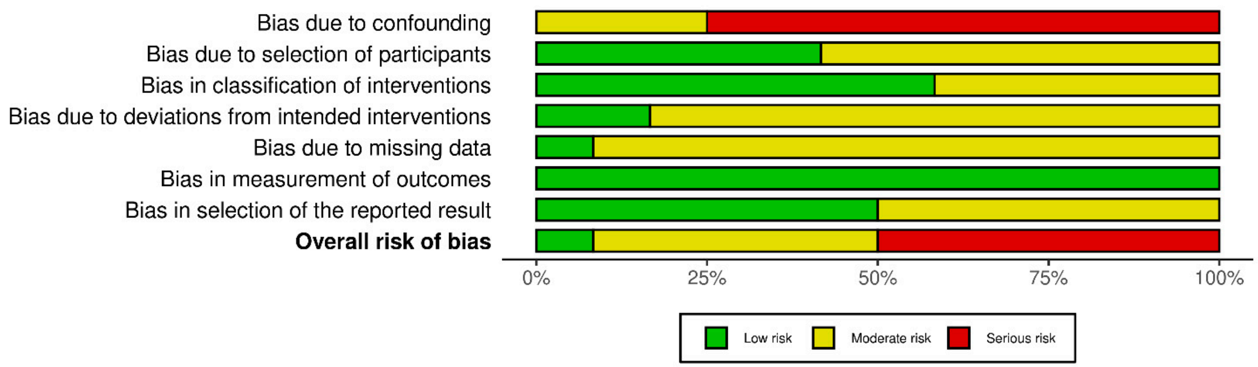

Supplementary Figure S2: Quality Assessment of Included Observational Studies Using the ROBINS-I Tool

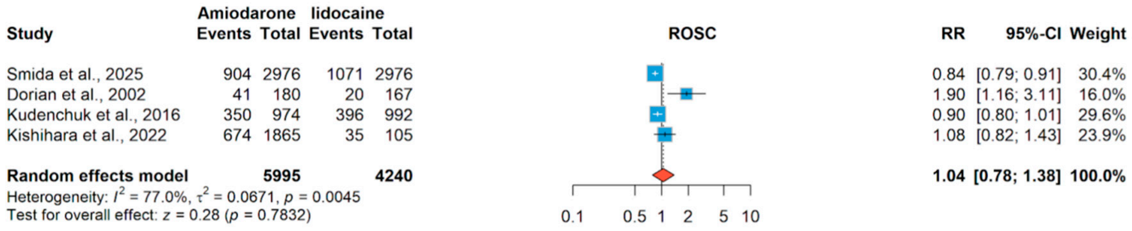

Supplementary Figure S3: Forest Plot of Return of Spontaneous Circulation in Out-Of-Hospital Cardiac Arrest (OHCA). Effect estimates compare amiodarone with lidocaine; RR < 1 favors lidocaine, whereas RR > 1 favors amiodarone.

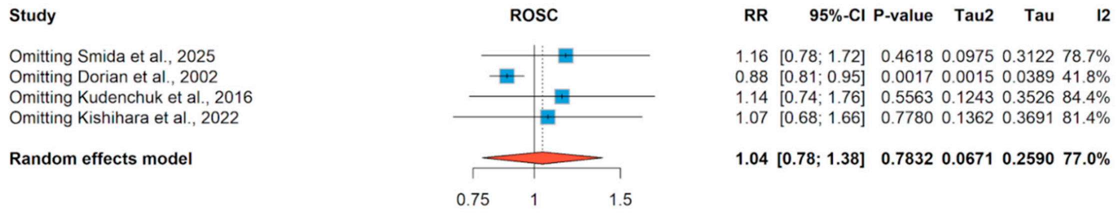

Supplementary Figure S4: Leave-One-Out Plot of Return of Spontaneous Circulation in Out-of-Hospital Cardiac Arrest (OHCA). Effect estimates compare amiodarone with lidocaine; RR < 1 favors lidocaine, whereas RR > 1 favors amiodarone.

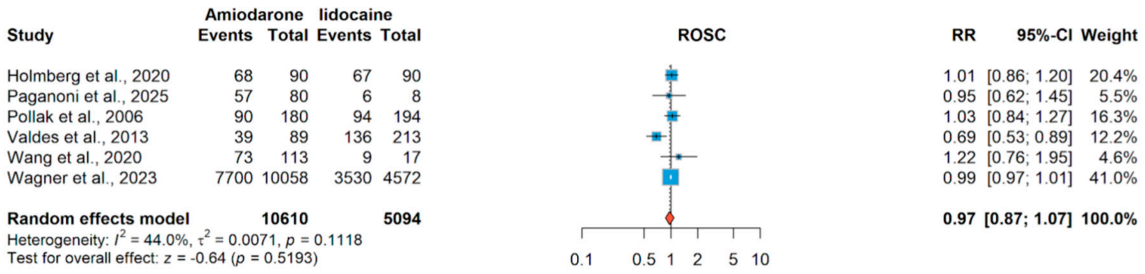

Supplementary Figure S5: Forest Plot of Return of Spontaneous Circulation in In-Hospital Cardiac Arrest (IHCA). Effect estimates compare amiodarone with lidocaine; RR < 1 favors lidocaine, whereas RR > 1 favors amiodarone.

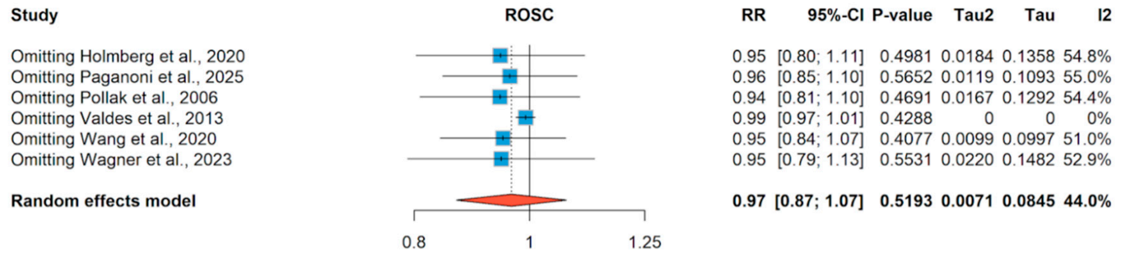

Supplementary Figure S6: Leave-One-Out Plot of Return of Spontaneous Circulation in In-Hospital Cardiac Arrest (IHCA). Effect estimates compare amiodarone with lidocaine; RR < 1 favors lidocaine, whereas RR > 1 favors amiodarone.

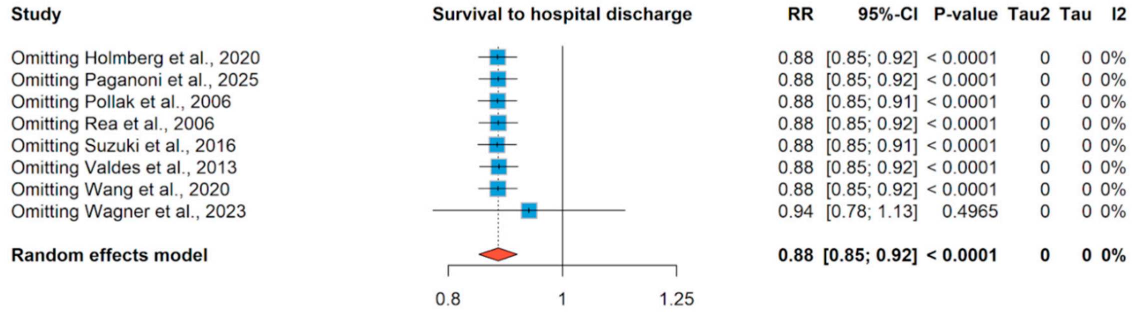

Supplementary Figure S7: Leave-One-Out Plot of Survival to Hospital Discharge in In-Hospital Cardiac Arrest (IHCA). Effect estimates compare amiodarone with lidocaine; RR < 1 favors lidocaine, whereas RR > 1 favors amiodarone.

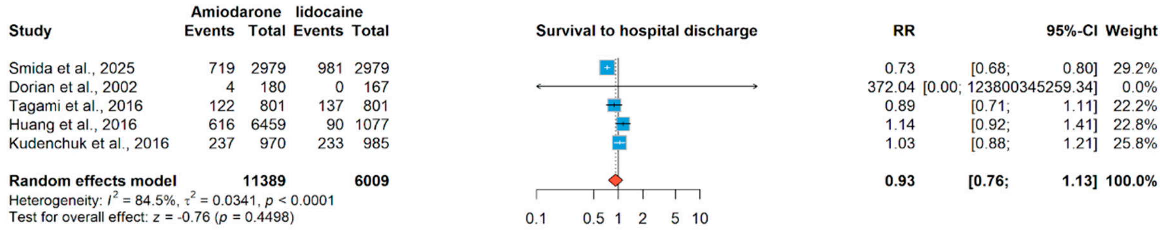

Supplementary Figure S8: Forest Plot of Survival to Hospital Discharge in Out-Of-Hospital Cardiac Arrest (OHCA). Effect estimates compare amiodarone with lidocaine; RR < 1 favors lidocaine, whereas RR > 1 favors amiodarone.

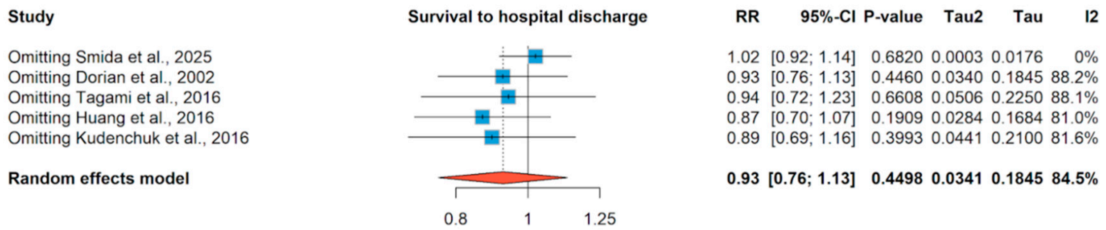

Supplementary Figure S9: Leave-One-Out Plot of Survival to Hospital Discharge in Out-Of-Hospital Cardiac Arrest (OHCA). Effect estimates compare amiodarone with lidocaine; RR < 1 favors lidocaine, whereas RR > 1 favors amiodarone.

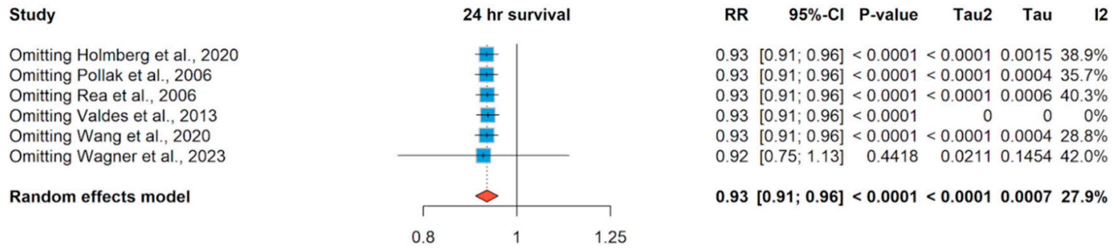

Supplementary Figure S10: Leave-One-Out Plot of 24-hour Survival. Effect estimates compare amiodarone with lidocaine; RR < 1 favors lidocaine, whereas RR > 1 favors amiodarone.

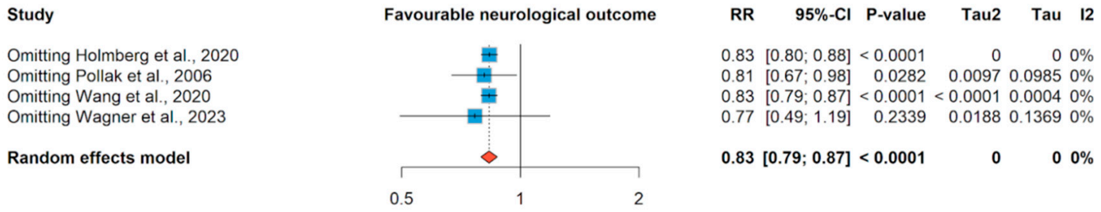

Supplementary Figure S11: Leave-One-Out Plot of Favourable Neurological Outcome. Effect estimates compare amiodarone with lidocaine; RR < 1 favors lidocaine, whereas RR > 1 favors amiodarone.

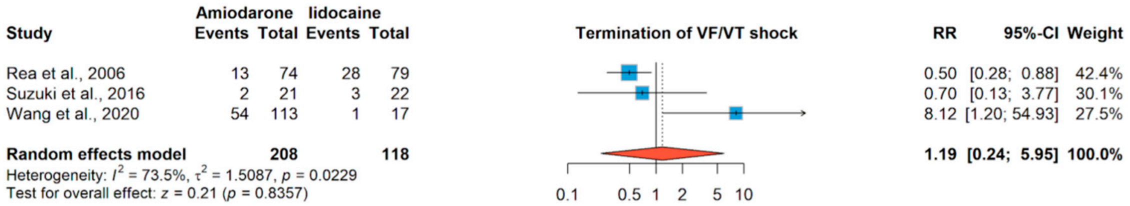

Supplementary Figure S12: Forest Plot of Termination of Ventricular Fibrillation/pulseless Ventricular Tachycardia. Effect estimates compare amiodarone with lidocaine; RR < 1 favors lidocaine, whereas RR > 1 favors amiodarone.

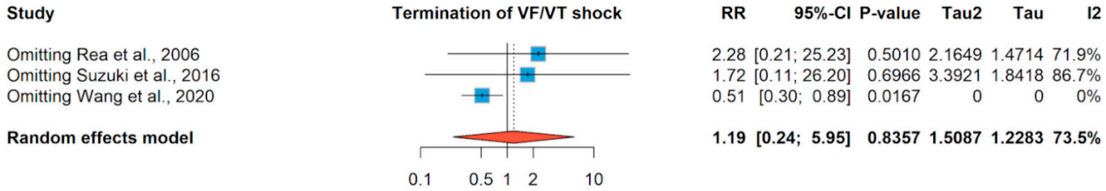

Supplementary Figure S13: Leave-One-Out Plot of Termination of Ventricular Fibrillation/pulseless Ventricular Tachycardia. Effect estimates compare amiodarone with lidocaine; RR < 1 favors lidocaine, whereas RR > 1 favors amiodarone.

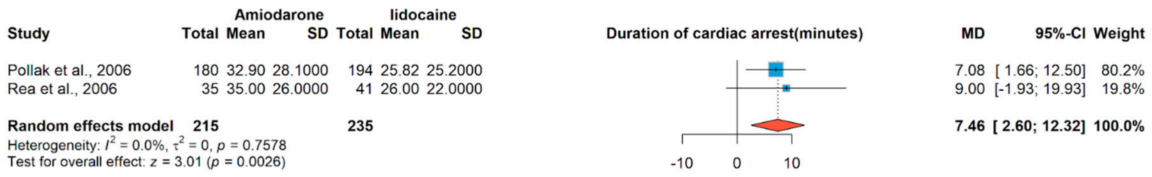

Supplementary Figure S14: Forest Plot of Duration of Cardiac Arrest (CA). Effect estimates compare amiodarone with lidocaine; MD > 0 indicates a longer cardiac-arrest duration with amiodarone, whereas MD < 0 indicates a longer duration with lidocaine.

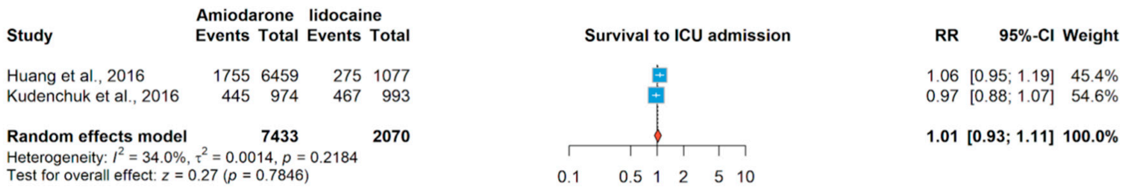

Supplementary Figure S15: Forest Plot of Survival to ICU Admission. Effect estimates compare amiodarone with lidocaine; RR < 1 favors lidocaine, whereas RR > 1 favors amiodarone.

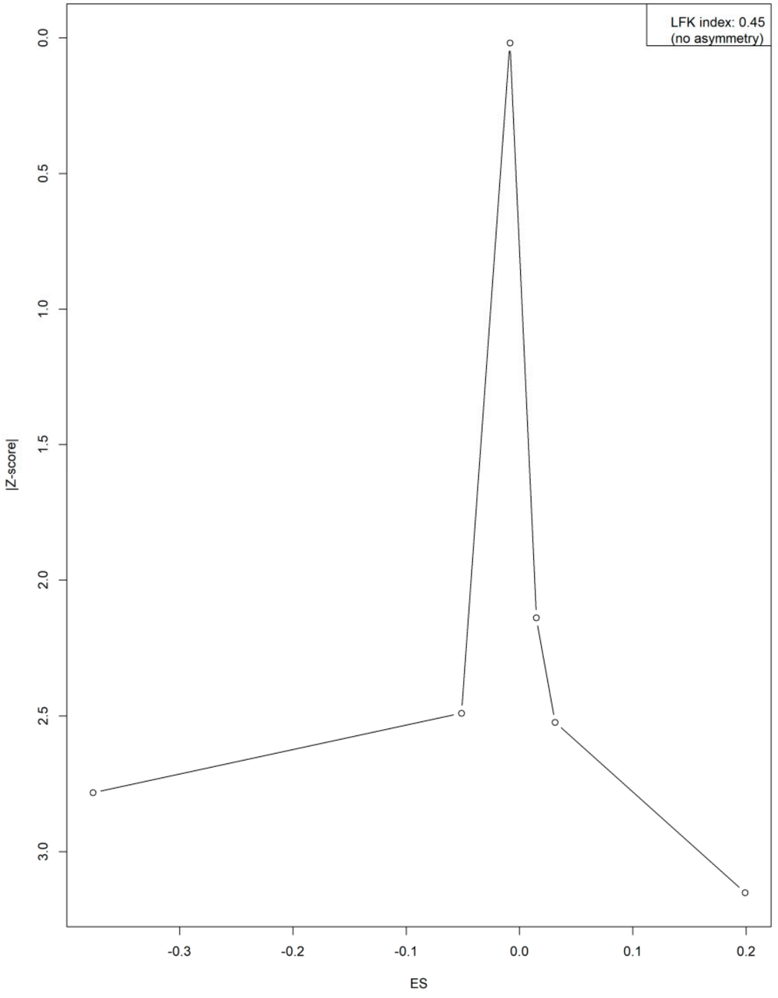

Supplementary Figure S16: LFK Plot of Return of Spontaneous Circulation in In-Hospital Cardiac Arrest (IHCA)

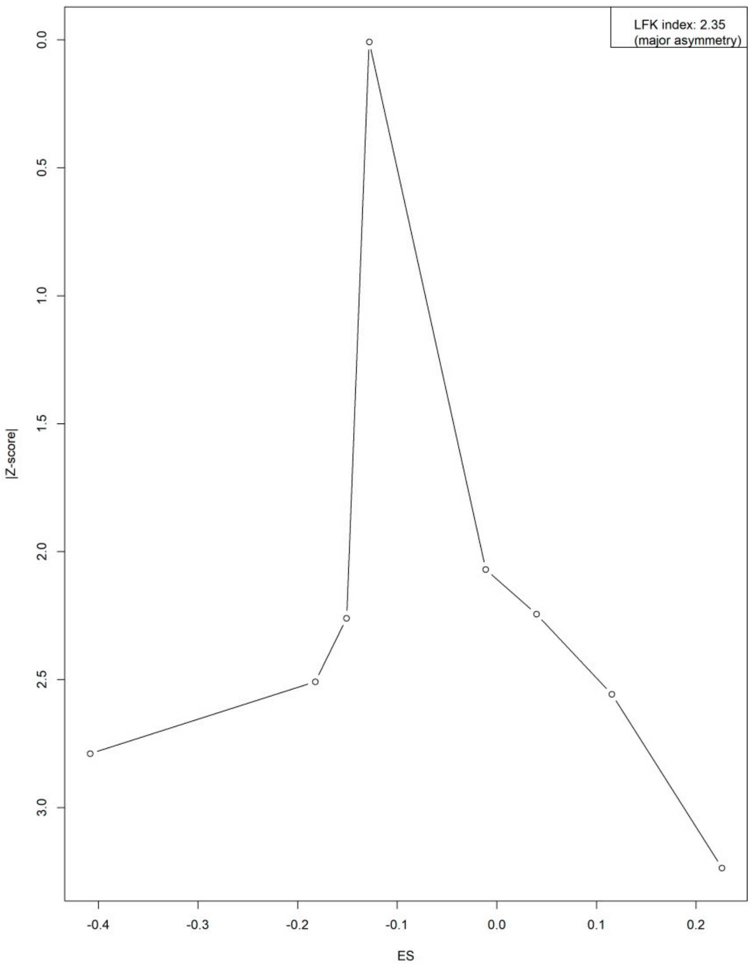

Supplementary Figure S17: LFK Plot of Survival to Hospital Discharge in In-Hospital Cardiac Arrest (IHCA)

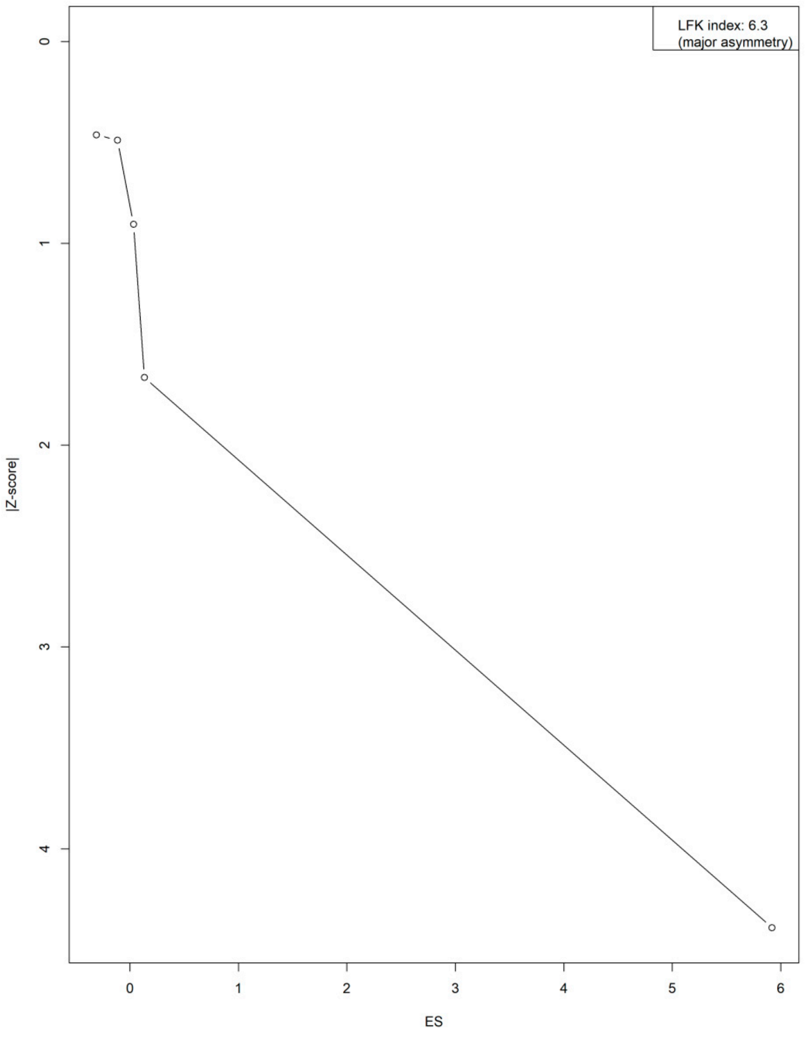

Supplementary Figure S18: LFK Plot of Survival to Hospital Discharge in Out-of-Hospital Cardiac Arrest (OHCA)

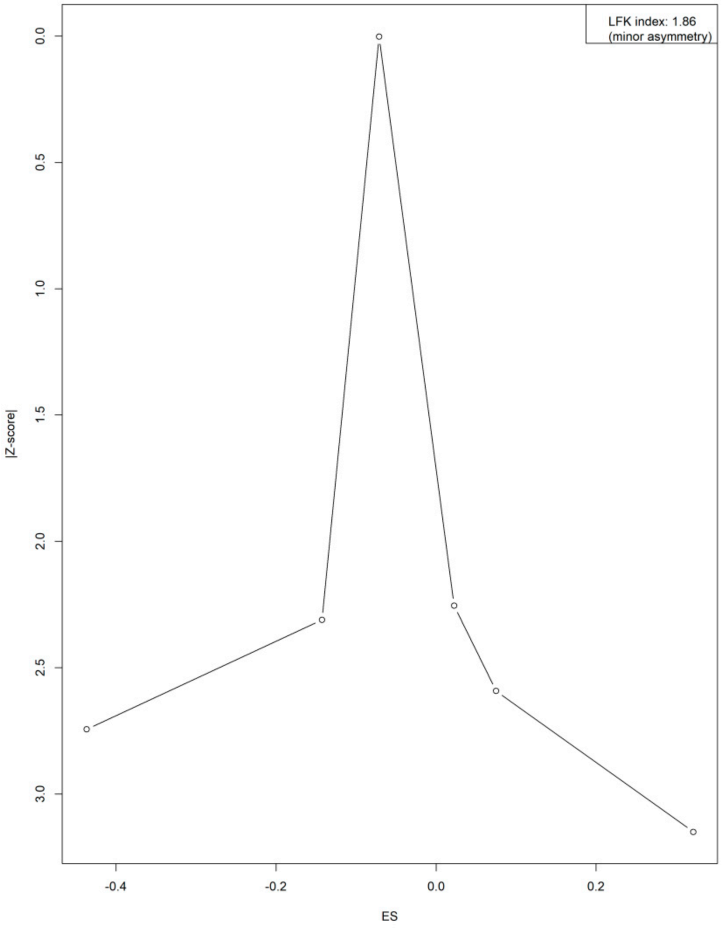

Supplementary Figure S19: LFK Plot of 24-hour Survival

Forest Plot: Risk Ratio (RR) — Return of Spontaneous Circulation

Study	Events(Amiodarone)	N(Amiodarone)	Events(Lidocaine)	N(Lidocaine)	RR	95% CrI
Holmberg et al., 2020	68	90	67	90	0.99	0.87 to 1.16
Paganoni et al., 2025	57	80	6	8	0.96	0.75 to 1.24
Pollak et al., 2006	90	180	94	194	1.00	0.85 to 1.19
Valdes et al., 2013	39	89	136	213	0.82	0.63 to 0.99
Wang et al., 2020	73	113	9	17	1.01	0.81 to 1.40
Wagner et al., 2023	7704	10058	3534	4572	0.99	0.97 to 1.01
Smida et al., 2025	904	2976	1071	2976	0.86	0.80 to 0.92
Dorian et al., 2002	41	180	20	167	1.10	0.87 to 1.72
Kudenchuk et al., 2016	350	974	396	992	0.91	0.82 to 1.01
Kishihara et al., 2022	674	1865	35	105	1.01	0.84 to 1.27
**Overall (Posterior Median)**					**0.96**	**0.86 to 1.11**
**Prediction Interval (Median)**					**0.96**	**0.64 to 1.47**
**Heterogeneity (tau, log)**					**0.13**	**0.04 to 0.31**

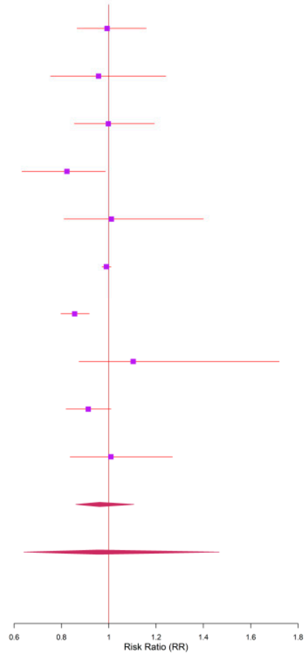

Supplementary Figure S20: Bayesian Forest plot for Overall ROSC

Forest Plot: Risk Ratio (RR) — In-Hospital Return of Spontaneous Circulation Subgroup

Study	Events(Amiodarone)	N(Amiodarone)	Events(Lidocaine)	N(Lidocaine)	RR	95% CrI
Holmberg et al., 2020	68	90	67	90	0.99	0.87 to 1.14
Paganoni et al., 2025	57	80	6	8	0.97	0.74 to 1.22
Pollak et al., 2006	90	180	94	194	0.99	0.86 to 1.18
Valdes et al., 2013	39	89	136	213	0.86	0.64 to 1.02
Wang et al., 2020	73	113	9	17	1.00	0.82 to 1.37
Wagner et al., 2023	7704	10058	3534	4572	0.99	0.97 to 1.01
**Overall (Posterior Median)**					**0.97**	**0.82 to 1.11**
**Prediction Interval (Median)**					**0.97**	**0.63 to 1.42**
**Heterogeneity (tau, log)**					**0.10**	**0.01 to 0.33**

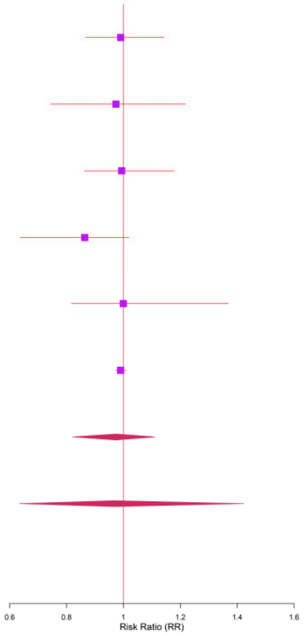

Supplementary Figure S21: Bayesian Forest plot for IHCA-ROSC subgroup

Forest Plot: Risk Ratio (RR) — Out-Hospital Return of Spontaneous Circulation Subgroup

Study	Events(Amiodarone)	N(Amiodarone)	Events(Lidocaine)	N(Lidocaine)	RR	95% CrI
Smida et al., 2025	904	2976	1071	2976	0.85	0.79 to 0.91
Dorian et al., 2002	41	180	20	167	1.27	0.86 to 2.21
Kudenchuk et al., 2016	350	974	396	992	0.91	0.81 to 1.01
Kishihara et al., 2022	674	1865	35	105	1.04	0.83 to 1.35
**Overall (Posterior Median)**					**0.99**	**0.78 to 1.41**
**Prediction Interval (Median)**					**0.98**	**0.54 to 1.99**
**Heterogeneity (tau, log)**					**0.21**	**0.02 to 0.52**

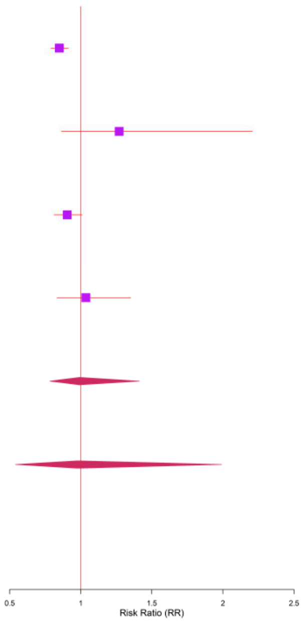

Supplementary Figure S22: Bayesian Forest plot for OHCA-ROSC subgroup

Forest Plot: Risk Ratio (RR) — 24-hr Survival in Hospiatal

Study	Events(Amiodarone)	N(Amiodarone)	Events(Lidocaine)	N(Lidocaine)	RR	95% CrI
Holmberg et al., 2020	45	90	44	90	0.94	0.80 to 1.19
Pollak et al., 2006	49	180	49	194	0.95	0.80 to 1.25
Rea et al., 2006	34	62	43	68	0.92	0.74 to 1.08
Valdes et al., 2013	27	89	100	213	0.87	0.61 to 1.01
Wang et al., 2020	55	113	6	17	0.95	0.77 to 1.40
Wagner et al., 2023	5937	10058	2898	4572	0.93	0.91 to 0.96
**Overall (Posterior Median)**					**0.93**	**0.79 to 1.09**
**Prediction Interval (Median)**					**0.92**	**0.59 to 1.46**
**Heterogeneity (tau, log)**					**0.09**	**0.00 to 0.34**

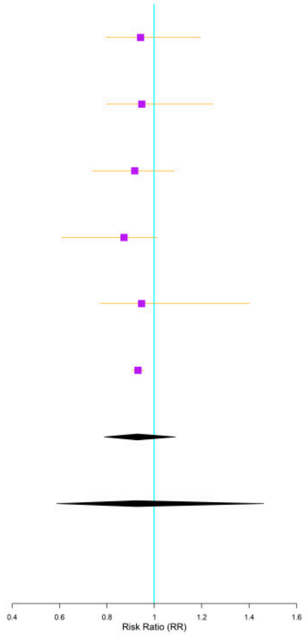

Supplementary Figure S23: Bayesian Forest plot for 24-hr survival in Hospital

Forest Plot: Risk Ratio (RR) — In-Hospital Favourable Neurologic Outcome

Study	Events(Amiodarone)	N(Amiodarone)	Events(Lidocaine)	N(Lidocaine)	RR	95% CrI
Holmberg et al., 2020	12	85	20	78	0.79	0.47 to 1.03
Pollak et al., 2006	21	180	24	194	0.84	0.62 to 1.22
Wang et al., 2020	13	113	2	17	0.83	0.52 to 1.29
Wagner et al., 2023	3083	10058	1681	4572	0.83	0.79 to 0.87
**Overall (Posterior Median)**					**0.82**	**0.60 to 1.06**
**Prediction Interval (Median)**					**0.81**	**0.37 to 1.70**
**Heterogeneity (tau, log)**					**0.12**	**0.01 to 0.44**

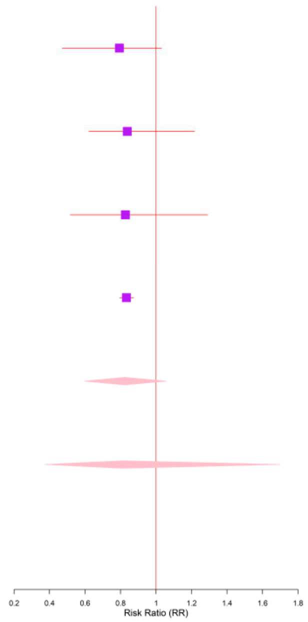

Supplementary Figure S24: Bayesian Forest plot for In-Hospital Favourable Neurological Outcome

Forest Plot: Risk Ratio (RR) — In-Hospital Termination of VT/VF with Shock

Study	Events(Amiodarone)	N(Amiodarone)	Events(Lidocaine)	N(Lidocaine)	RR	95% CrI
Rea et al., 2006	13	74	28	79	0.61	0.35 to 1.02
Suzuki et al., 2016	2	21	3	22	0.68	0.32 to 1.76
Wang et al., 2020	54	113	1	17	0.79	0.39 to 2.97
**Overall (Posterior Median)**					**0.70**	**0.36 to 1.54**
**Prediction Interval (Median)**					**0.69**	**0.10 to 4.81**
**Heterogeneity (tau, log)**					**0.21**	**0.01 to 0.64**

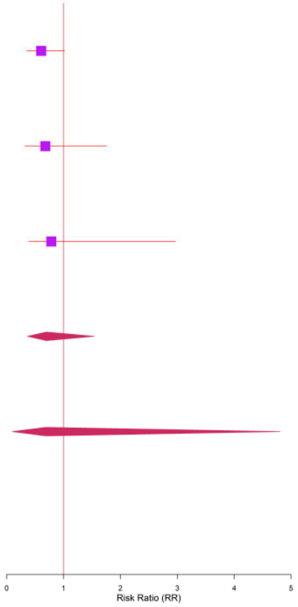

Supplementary Figure S25: Bayesian Forest plot for In-Hospital termination of VF/pVT with shock

Forest Plot: Risk Ratio (RR) — Out-Hospital Survival to ICU Admission

Study	Events(Amiodarone)	N(Amiodarone)	Events(Lidocaine)	N(Lidocaine)	RR	95% CrI
Huang et al., 2016	1755	6459	275	1077	1.04	0.95 to 1.16
Kudenchuk et al., 2016	445	974	467	993	0.99	0.89 to 1.07
**Overall (Posterior Median)**					**1.01**	**0.77 to 1.33**
**Prediction Interval (Median)**					**1.01**	**0.63 to 1.62**
**Heterogeneity (tau, log)**					**0.10**	**0.00 to 0.44**

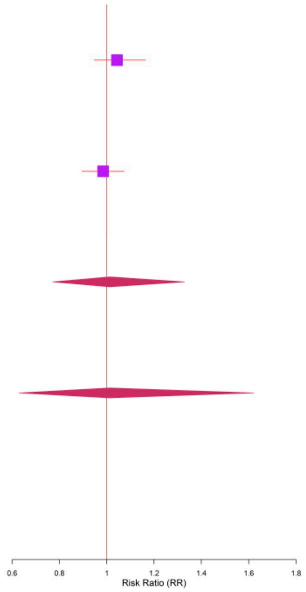

Supplementary Figure S26: Bayesian Forest plot for OHCA survival to ICU

Forest Plot: Risk Ratio (RR) — Survival to Hospital Discharge

Study	Events(Amiodarone)	N(Amiodarone)	Events(Lidocaine)	N(Lidocaine)	RR	95% CrI
Holmberg et al., 2020	28	90	28	89	0.93	0.73 to 1.23
Paganoni et al., 2025	25	80	3	8	0.91	0.67 to 1.26
Pollak et al., 2006	28	180	29	194	0.94	0.73 to 1.26
Rea et al., 2006	29	74	36	79	0.90	0.71 to 1.13
Suzuki et al., 2016	15	21	14	22	0.97	0.77 to 1.30
Valdes et al., 2013	15	89	54	213	0.86	0.63 to 1.10
Wang et al., 2020	25	113	3	17	0.93	0.69 to 1.32
Wagner et al., 2023	4196	10058	2168	4572	0.88	0.85 to 0.92
Smida et al., 2025	719	2979	981	2979	0.75	0.69 to 0.81
Dorian et al., 2002	4	181	0	168	0.92	0.68 to 1.38
Tagami et al., 2016	122	801	137	801	0.90	0.75 to 1.07
Huang et al., 2016	616	6459	90	1077	1.04	0.88 to 1.27
Kudenchuk et al., 2016	237	970	233	985	1.00	0.87 to 1.16
**Overall (Posterior Median)**					**0.92**	**0.81 to 1.05**
**Prediction Interval (Median)**					**0.91**	**0.54 to 1.56**
**Heterogeneity (tau, log)**					**0.14**	**0.07 to 0.28**

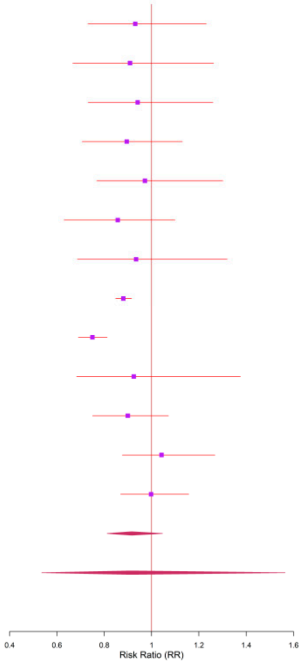

Supplementary Figure S27: Bayesian Forest plot for overall survival to hospital discharge

Forest Plot: Risk Ratio (RR) — Out-Hospital Survival to Hospital Discharge Subgroup

Study	Events(Amiodarone)	N(Amiodarone)	Events(Lidocaine)	N(Lidocaine)	RR	95% CrI
Smida et al., 2025	719	2979	981	2979	0.74	0.68 to 0.81
Dorian et al., 2002	4	181	0	168	0.97	0.60 to 1.80
Tagami et al., 2016	122	801	137	801	0.90	0.74 to 1.09
Huang et al., 2016	616	6459	90	1077	1.08	0.89 to 1.33
Kudenchuk et al., 2016	237	970	233	985	1.01	0.87 to 1.19
**Overall (Posterior Median)**					**0.93**	**0.74 to 1.23**
**Prediction Interval (Median)**					**0.93**	**0.52 to 1.70**
**Heterogeneity (tau, log)**					**0.20**	**0.09 to 0.46**

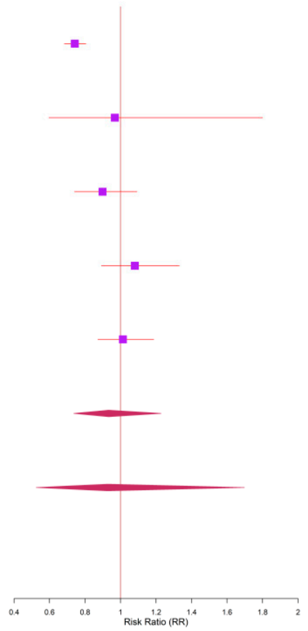

Supplementary Figure S28: Bayesian Forest plot for Out-Hospital Survival to hospital discharge subgroup

Forest Plot: Risk Ratio (RR) — In-Hospital Survival to Hospital Discharge Subgroup

Study	Events(Amiodarone)	N(Amiodarone)	Events(Lidocaine)	N(Lidocaine)	RR	95% CrI
Holmberg et al., 2020	28	90	28	89	0.90	0.75 to 1.16
Paganoni et al., 2025	25	80	3	8	0.89	0.69 to 1.18
Pollak et al., 2006	28	180	29	194	0.90	0.76 to 1.20
Rea et al., 2006	29	74	36	79	0.89	0.73 to 1.10
Suzuki et al., 2016	15	21	14	22	0.91	0.78 to 1.23
Valdes et al., 2013	15	89	54	213	0.88	0.65 to 1.07
Wang et al., 2020	25	113	3	17	0.90	0.72 to 1.24
Wagner et al., 2023	4196	10058	2168	4572	0.88	0.85 to 0.91
**Overall (Posterior Median)**					**0.89**	**0.79 to 1.05**
**Prediction Interval (Median)**					**0.89**	**0.53 to 1.53**
**Heterogeneity (tau, log)**					**0.07**	**0.00 to 0.28**

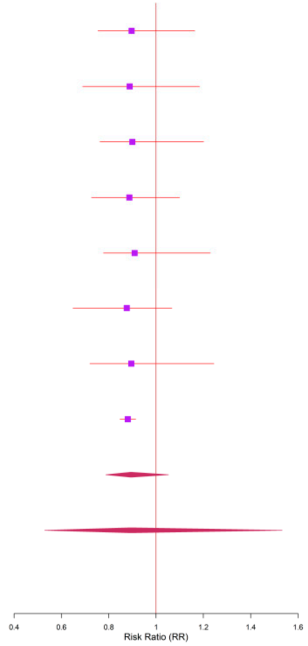

Supplementary Figure S29: Bayesian Forest plot for In-Hospital survival to hospital discharge subgroup

Study	Amiodarone Events	Amiodarone Total	Lidocaine Events	Lidocaine Total	RR	95%-CI	Weight
**Adults**							
Paganoni et al., 2025	25	80	3	8	0.83	[0.32; 2.16]	0.2%
Pollak et al., 2006	28	180	29	194	1.04	[0.65; 1.68]	0.6%
Rea et al., 2006	29	74	36	79	0.86	[0.59; 1.25]	1.0%
Suzuki et al., 2016	15	21	14	22	1.12	[0.74; 1.70]	0.8%
Wang et al., 2020	25	113	3	17	1.25	[0.42; 3.71]	0.1%
Wagner et al., 2023	4196	10058	2168	4572	0.88	[0.85; 0.91]	96.0%
**Random effects model**		**10526**		**4892**	**0.88**	**[0.85; 0.92]**	**98.7%**
Heterogeneity: $I^2 = 0\%$, $\tau^2 = 0$, $p = 0.8205$							
**Pediatrics**							
Holmberg et al., 2020	28	90	28	89	0.99	[0.64; 1.53]	0.7%
Valdes et al., 2013	15	89	54	213	0.66	[0.40; 1.11]	0.5%
**Random effects model**		**179**		**302**	**0.83**	**[0.57; 1.22]**	**1.3%**
Heterogeneity: $I^2 = 25\%$, $\tau^2 = 0.0197$, $p = 0.2483$							
**Random effects model**		**10705**		**5194**	**0.88**	**[0.85; 0.92]**	**100.0%**
Heterogeneity: $I^2 = 0.0\%$, $\tau^2 = 0$, $p = 0.8219$							
Test for overall effect: $z = -6.56$ ($p < 0.0001$)							
Test for subgroup differences: $\chi^2_1 = 0.09$, df = 1 ($p = 0.7640$)							

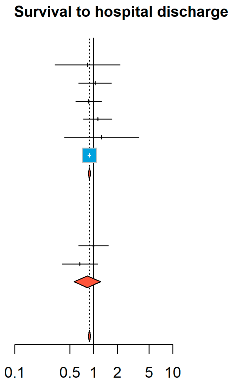

Supplementary Figure S30: Forest Plot of Adult versus Pediatric Subgroup Analysis for In-Hospital Survival to Hospital Discharge. Effect estimates compare amiodarone with lidocaine; RR < 1 favors lidocaine, whereas RR > 1 favors amiodarone.

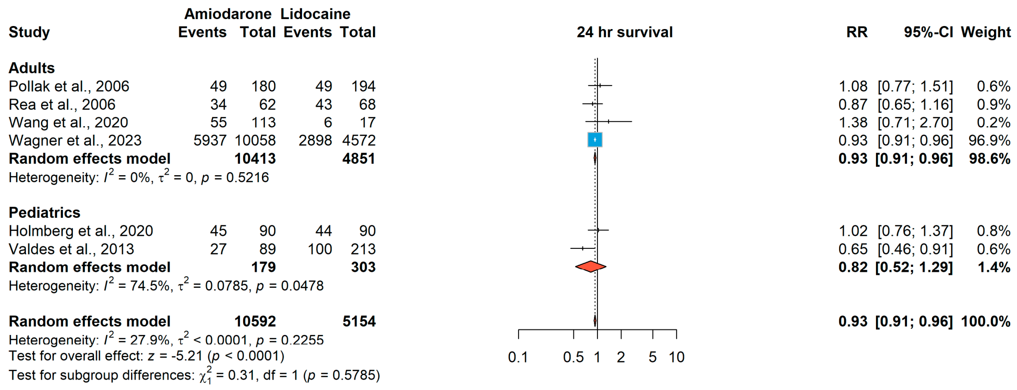

Supplementary Figure S31: Forest Plot of Adult versus Pediatric Subgroup Analysis for In-Hospital 24-Hour Survival. Effect estimates compare amiodarone with lidocaine; RR < 1 favors lidocaine, whereas RR > 1 favors amiodarone.

Study	Amiodarone Events	Amiodarone Total	Lidocaine Events	Lidocaine Total	RR	95%-CI	Weight
**Adults**							
Pollak et al., 2006	21	180	24	194	0.94	[0.54; 1.63]	0.8%
Wang et al., 2020	13	113	2	17	0.98	[0.24; 3.96]	0.1%
Wagner et al., 2023	3083	10058	1681	4572	0.83	[0.79; 0.87]	98.6%
**Random effects model**		**10351**		**4783**	**0.83**	**[0.80; 0.88]**	**99.5%**
Heterogeneity: $I^2 = 0\%$, $\tau^2 = 0$, $p = 0.8864$							
**Pediatrics**							
Holmberg et al., 2020	12	85	20	78	0.55	[0.29; 1.05]	0.5%
**Random effects model**		**10436**		**4861**	**0.83**	**[0.79; 0.87]**	**100.0%**

Heterogeneity: $I^2 = 0.0\%$, $\tau^2 = 0$, $p = 0.6098$
Test for overall effect: $z = -7.52$ ($p < 0.0001$)
Test for subgroup differences: $\chi^2_1 = 1.58$, df = 1 ($p = 0.2084$)

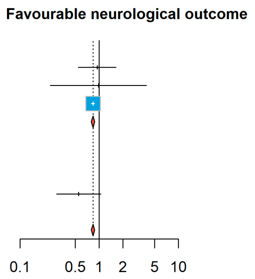

Supplementary Figure S32: Forest Plot of Adult versus Pediatric Subgroup Analysis for In-Hospital Favorable Neurological Outcome. Effect estimates compare amiodarone with lidocaine; RR < 1 favors lidocaine, whereas RR > 1 favors amiodarone.

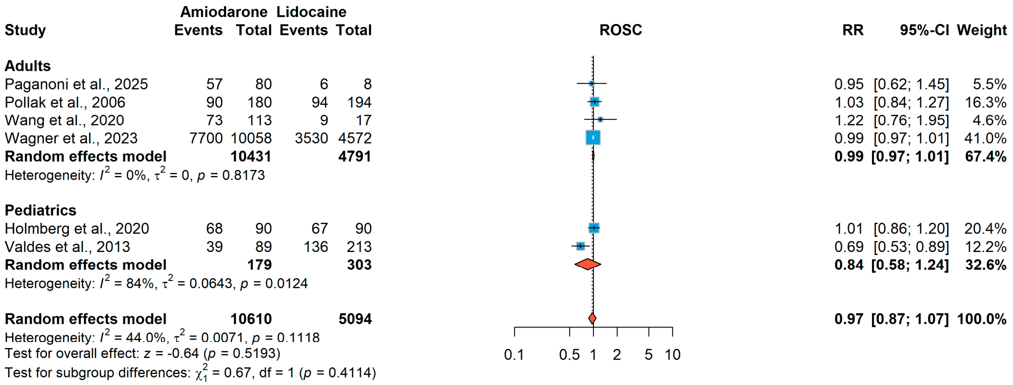

Supplementary Figure S33: Forest Plot of Adult versus Pediatric Subgroup Analysis for Return of Spontaneous Circulation (ROSC) in In-Hospital Cardiac Arrest. Effect estimates compare amiodarone with lidocaine; RR < 1 favors lidocaine, whereas RR > 1 favors amiodarone.

Supplementary Table S3. GRADE assessment of the certainty of evidence for amiodarone versus lidocaine in patients with shockable cardiac arrest

Setting	Outcome	Studies, n	Participants, n	Effect estimate (95% CI)	Risk of bias	Inconsistency	Indirectness	Imprecision	Publication bias	Certainty of evidence u
**IHCA**	Return of spontaneous circulation (ROSC)	6 (0 RCT, 6 NRS)[r]	15,704	RR 0.97 (0.87–1.07)	**Very serious[a]**	**Serious[b]**	Not serious	Not serious	Not serious	**⊕◯◯◯ Very low**
**IHCA**	Survival to hospital discharge	8 (0 RCT, 8 NRS)[r]	15,899	RR 0.88 (0.85–0.92)	**Very serious[a]**	Not serious	Not serious	Not serious	**Serious[c]**	**⊕◯◯◯ Very low**
**IHCA**	24-hour survival	6 (0 RCT, 6 NRS)[r]	15,746	RR 0.93 (0.91–0.96)	**Very serious[a]**	Not serious	Not serious	Not serious	Not serious[d]	**⊕⊕◯◯ Low**
**IHCA**	Favourable neurological outcome	4 (0 RCT, 4 NRS)[r]	15,297	RR 0.83 (0.79–0.87)	**Very serious[a]**	Not serious	Not serious	Not serious	Not assessable[e]	**⊕⊕◯◯ Low**
**IHCA**	Termination of VF/pVT	3 (0 RCT, 3 NRS)[r]	326	RR 1.19 (0.24–5.95)	**Very serious[f]**	**Serious[g]**	Not serious	**Very serious[h]**	Not assessable[e]	**⊕◯◯◯ Very low**
**IHCA**	Duration of cardiac arrest	2 (0 RCT, 2 NRS)[r]	450	MD +7.46 min (2.60–12.32)	**Very serious[i]**	Not serious	Not serious	Not serious[j]	Not assessable[e]	**⊕⊕◯◯ Low**
**OHCA**	Return of spontaneous circulation (ROSC)	4 (2 RCT, 2 NRS)[s]	10,235	RR 1.04 (0.78–1.38)	**Serious[k]**	**Serious[l]**	Not serious	**Serious[m]**	Not assessable[e]	**⊕◯◯◯ Very low**
**OHCA**	Survival to hospital discharge	5 (2 RCT, 3 NRS)[s]	17,398	RR 0.93 (0.76–1.13)	**Serious[k]**	**Serious[n]**	Not serious	**Serious[o]**	**Serious[p]**	**⊕◯◯◯ Very low**
**OHCA**	Survival to ICU admission	2 (1 RCT, 1 NRS)[s]	9,503	RR 1.01 (0.93–1.11)	**Serious[q]**	Not serious	Not serious	Not serious	Not assessable[e]	**⊕⊕⊕◯ Moderate**

Footnotes

[a] Downgraded two levels for very serious risk of bias because the IHCA evidence was overwhelmingly derived from observational studies at serious ROBINS-I risk, principally because of confounding. For several outcomes, the large Wagner et al. cohort contributed approximately 96–99% of the statistical weight. The manuscript also acknowledges important residual confounding related to disease severity, drug timing, defibrillation attempts, CPR characteristics, and comorbidities.

[b] Downgraded one level for inconsistency because moderate heterogeneity was present (I^2=44%) with variability in study-specific estimates. The manuscript reports RR 0.97 (95% CI 0.87–1.07) with I^2=44% for IHCA ROSC.

[c] Downgraded one level for suspected publication bias because major Doi-plot asymmetry was observed for IHCA survival to hospital discharge (LFK=2.35).

[d] No downgrade for publication bias. The Doi plot showed only minor asymmetry; this was not considered sufficient evidence to reduce certainty.

[e] Publication bias was not downgraded because fewer than five studies contributed to the outcome, preventing a reliable formal assessment. Failure to perform a statistical test with few studies does not itself constitute evidence of publication bias.

[f] Downgraded two levels for risk of bias because approximately 70% of the statistical weight was derived from studies assessed at serious ROBINS-I risk and the remainder from a study at moderate risk.

[g] Downgraded one level for serious inconsistency because heterogeneity was substantial (I^2=73.5%) and study estimates differed markedly in magnitude and direction. Exclusion of Wang et al. reduced I^2 to 0% and changed the pooled estimate to RR 0.51 (95% CI 0.30–0.89).

[h] Downgraded two levels for very serious imprecision because only 326 participants contributed and the very wide CI (RR 0.24–5.95) encompassed substantial effects favoring either intervention. GRADE recommends downgrading when CIs encompass clinically important benefit and harm.

[i] Downgraded two levels for very serious risk of bias because both studies contributing to cardiac-arrest duration were observational studies assessed at serious ROBINS-I risk.

[j] No downgrade for imprecision because the CI remained entirely on the side of longer cardiac-arrest duration with amiodarone (MD 7.46 minutes, 95% CI 2.60–12.32). However, no prespecified minimal important difference was reported, and this judgement should be reconsidered if a specific clinical threshold is adopted.

[k] Downgraded one level for risk of bias because the OHCA syntheses combined low-risk randomized evidence with a substantial contribution from observational studies at moderate ROBINS-I risk.

[l] Downgraded one level for serious inconsistency because heterogeneity was substantial (I^2=77%). Leave-one-out analysis showed important sensitivity to Dorian et al.; its exclusion changed the pooled estimate to RR 0.88 (95% CI 0.81–0.95) and reduced heterogeneity.

[m] Downgraded one level for imprecision because the 95% CI for OHCA ROSC (RR 0.78–1.38) included potentially important effects favoring either treatment. GRADE notes that CIs including appreciable benefit or harm warrant consideration for downgrading.

[n] Downgraded one level for serious inconsistency because heterogeneity for OHCA survival to hospital discharge was very high (I^2=84.5%). Leave-one-out analysis showed that removal of Smida et al. reduced heterogeneity to approximately 0%, indicating substantial influence of an individual study.

[o] Downgraded one level for imprecision because the CI (RR 0.76–1.13) encompassed potentially clinically important survival differences in both directions.

[p] Downgraded one level for suspected publication bias because major Doi-plot asymmetry was observed for OHCA survival to hospital discharge (LFK=6.3).

[q] Downgraded one level for risk of bias. Kudenchuk et al. was a low-risk RCT, whereas the supplied ROBINS-I figure for Huang et al. shows **moderate risk in the confounding domain (D1)**. Therefore we used the conservative outcome-level GRADE judgement of serious risk of bias rather than retaining high certainty. The pooled estimate was RR 1.01 (95% CI 0.93–1.11; I^2=34%).

[r] Evidence base: informed exclusively by non-randomised studies. Both randomised trials included in this review (Dorian et al. 2002 and Kudenchuk et al. 2016) enrolled out-of-hospital populations, so no randomised evidence contributes to any in-hospital outcome. The contributing cohorts were Holmberg 2020, Paganoni 2025, Pollak 2006, Rea 2006, Suzuki 2016, Valdes 2013, Wang 2020 and Wagner 2023, in the combinations shown for each outcome.

[s] Evidence base: mixed randomised and non-randomised evidence. For OHCA ROSC, two RCTs (Dorian 2002, Kudenchuk 2016) contributed approximately 43% of the subgroup weight alongside two cohorts (Smida 2025, Kishihara 2022). For OHCA survival to hospital discharge, two RCTs contributed alongside three cohorts (Smida 2025, Tagami 2016, Huang 2016), although the randomised contribution is effectively confined to Kudenchuk (14.1% of the 54.1% subgroup weight) because Dorian received 0.0% weight, no events having been recorded in its lidocaine arm. For OHCA survival to ICU admission, one RCT (Kudenchuk 2016) and one cohort (Huang 2016) each contributed approximately half of the weight. Risk-of-bias judgements for these outcomes (footnotes [k] and [q]) reflect this mixed composition, and were downgraded one rather than two levels accordingly.

[u] Starting certainty and rating up. Consistent with GRADE guidance for reviews applying ROBINS-I, bodies of evidence from non-randomised studies were started at high certainty rather than low, with confounding and other design limitations captured within the risk-of-bias domain; randomised bodies were likewise started at high. All judgements in this table are therefore downgrades from high certainty. Rating up was formally considered for every outcome and applied to none: pooled effects were modest (all RRs between 0.83 and 1.19, none reaching the threshold for a large effect), dosing was not reported in a form permitting assessment of a dose–response gradient, and plausible residual confounding would be expected to exaggerate rather than attenuate the apparent harm associated with amiodarone, since patients with more refractory arrests were more likely to receive it.

Legend. *GRADE, Grading of Recommendations Assessment, Development and Evaluation; NRS, non-randomised study; RCT, randomised controlled trial; IHCA, in-hospital cardiac arrest; OHCA, out-of-hospital cardiac arrest; ROSC, return of spontaneous circulation; VF, ventricular fibrillation; pVT, pulseless ventricular tachycardia; ICU, intensive care unit; RR, risk ratio; MD, mean difference; CI, confidence interval; ROBINS-I, Risk Of Bias In Non-randomised Studies of Interventions; RoB 2, Cochrane Risk of Bias 2 tool; LFK, Luis Furuya-Kanamori index.*

Certainty of evidence was classified as high (⨁⨁⨁⨁), moderate (⨁⨁⨁◯), low (⨁⨁◯◯), or very low (⨁◯◯◯). GRADE considers risk of bias, inconsistency, indirectness, imprecision, and publication bias for each outcome separately.

Supplementary Table S4. Study-Level Reporting of Potential Sources of Clinical Heterogeneity

A. In-hospital cardiac arrest (IHCA) studies

Study	Population	Antiarrhythmic timing	Shocks before drug / shock burden	Witnessed arrest	Bystander CPR	Epinephrine use	Baseline severity / arrest characteristics
**Pollak et al., 2006**	Adult IHCA	NR; (insufficient data)	NR	NR	N/A	NR	Pulseless VT/VF; arrest duration 32.9 ± 28.1 min with amiodarone vs 25.8 ± 25.2 min with other antiarrhythmics.
**Rea et al., 2006**	Adult IHCA	First antiarrhythmic: 6 ± 5 min with	first defibrillation	94% lidocaine vs	N/A	NR	Pulseless VT/VF; code duration 26 ± 22

		lidocaine vs 14 ± 9 min with amiodarone; combination therapy 9 ± 8 min.	6 ± 12 vs 8 ± 8 min.	89% amiodarone.			min with lidocaine vs 35 ± 26 min with amiodarone
**Paganoni et al., 2025**	Adult IHCA	Median 6 min (IQR 4–10)	Total 3 shocks (IQR 1–5); before-second-shock group 1 (1–4), after-second-shock group 4 (3–6).	NR	N/A	NR	VF 54.5%, pVT 38.6%; HF 48.9%, CAD 64.8%, CKD 44.3%.
**Holmberg et al., 2020**	Pediatric IHCA (≤18 years)	NR	Exact pre-drug number is not reported; sensitivity analyses considered ≥1 and ≥2 defibrillations.	99% lidocaine vs 94% amiodarone.	N/A	Epinephrine <2 min: 46% vs 46%; remaining patients received it >2 min or not at all.	Initial shockable rhythm 69% lidocaine vs 59% amiodarone; subsequent shockable rhythm 31% vs 41%
**Valdes et al., 2014**	Pediatric IHCA	Specific timing NR.	Mean 3 ± 4 defibrillation attempts.	96% overall.	N/A	Mean 5 ± 4 epinephrine doses.	pVT/VF; 42% had subsequent rather than initial pVT/VF; mean event/CPR duration 35 ± 36 min; illness category and pre-existing cardiorespiratory support were recorded.
**Suzuki et al., 2016**	Predominantly adult hospital cohort	exact resuscitation timing NR.	NR; electrical cardioversion was used in	NR	NR	NR	Heterogeneous ventricular-arrhythmia

			90.5% of the amiodarone group vs 77.3% of the lidocaine group.				cohort receiving continuous antiarrhythmic infusion after ROSC; LVEF 39–40%; prehospital cardiopulmonary arrest 4.8% vs 45.5%.
**Wang et al., 2020**	Adult IHCA	Median 9 min (IQR 4–16) overall; 9 (5–16) min with amiodarone vs 9 (4–16) min with lidocaine.	Shock-refractory VF/pVT requiring >1 shock; total shocks 4 (2–6) vs 5 (4–7); studies reported most drugs were likely administered after the third shock.	67.3% amiodarone vs 100% lidocaine.	N/A	NR	Shock-refractory VF/pVT; CCI 2.9; hypotension 25%; CPR duration 32 (19–45) vs 47 (29–96) min.
**Wagner et al., 2023**	Adult IHCA	NR	Pre-drug shock number NR; time to first defibrillation 2.4 ± 4.2 min with amiodarone vs 2.2 ± 3.9 min with lidocaine.	87.5% amiodarone vs 87.7% lidocaine.	N/A	Specific epinephrine use NR.	66% cardiac illness category; substantial renal/respiratory disease, sepsis, and vasopressor use; CPR duration and APACHE II were unavailable.

B. Out-of-hospital cardiac arrest (OHCA) studies

Study	Population	Antiarrhythmic timing	Shocks before drug /	Witnessed arrest	Bystander CPR	Epinephrine use	Baseline severity /

			shock burden				arrest characteristics
**Dorian et al., 2002 (ALIVE)**	Adult OHCA	25 ± 8 min with amiodarone vs 24 ± 7 min with lidocaine	Shock-resistant pathway: 3 shocks → epinephrine → fourth shock → study drug; recurrent VF was also eligible.	76% amiodarone vs 78% lidocaine.	26% vs 28%.	At least one IV epinephrine dose was required by protocol for the shock-resistant pathway.	initial VF 78–79%; cardiac disease 60%; some patients had transient ROSC before study-drug administration.
**Kudenchuk et al., 2016 (ALPS)**	Adult OHCA	Non-EMS-witnessed arrest: 19.3 ± 7.1 vs 19.3 ± 7.6 min from initial call; EMS-witnessed arrest: 11.7 ± 5.8 vs 12.1 ± 6.6 min from arrest.	Median 3 shocks (IQR 2–4) before the first trial drug in both groups.	EMS witnessed 6.0% vs 4.6%; bystander witnessed 65.4% vs 65.9%.	61.4% amiodarone vs 59.2% lidocaine.	98.7% vs 98.8% received epinephrine.	Initial shock-refractory VF/pVT after ≥1 shock; randomized groups were otherwise well balanced.
**Tagami et al., 2016**	Adult cardiogenic OHCA	exact arrest-to-drug time NR.	exact number NR.	NR	NR	87.4% amiodarone vs 86.9% lidocaine after matching.	Refractory VF/pVT on hospital arrival; adult cardiogenic OHCA; ECMO ~10–12% after matching.
**Huang et al., 2017**	Adult OHCA	Exact timing unavailable	Patient-specific pre-drug count NR	NR	NR	97.83% amiodarone vs 96.66% lidocaine; mean epinephrine dose 12.38 vs 12.09 mg.	Unstable adult shockable OHCA; CCI 4.57 vs 4.83; prehospital details were incompletely available.
**Kishihara et al., 2022**	Adult OHCA	No data available	Exact pre-drug number NR; shock	EMS witnessed 40.8% vs 48.6%;	48.3% amiodarone vs 43.8% lidocaine.	89.2% vs 83.8% before matching.	Heterogeneous initial rhythms despite subsequent

			delivery 95.6% amiodarone vs 79.1% lidocaine before matching.	other witnessed 32.2% vs 23.8%.			shockable rhythm: VF 62.2% vs 46.7%; PEA/asystole also present.
**Smida et al., 2025**	Adult OHCA	Time from scene arrival to first antiarrhythmic: 12.2 min amiodarone vs 11.5 min lidocaine before matching; 11.9 vs 11.5 min after matching.	Pre-drug shocks: 4 (IQR 2–6) vs 3 (2–4) before matching; after matching 4 (2–4) vs 3 (2–4).	EMS-witnessed arrests were excluded; among remaining arrests, witnessed 66.4% vs 67.7%.	Pre-EMS CPR 39.2% vs 41.3%.	NR	Initial shockable rhythm; ~93% presumed cardiac etiology; EMS-witnessed and pre-EMS ROSC cases were excluded.

Footnotes: NR = not reported; N/A = not applicable; CPR = cardiopulmonary resuscitation; VF = ventricular fibrillation; pVT = pulseless ventricular tachycardia; ROSC = return of spontaneous circulation; EMS = emergency medical services; CCI = Charlson Comorbidity Index; HF = heart failure; CAD = coronary artery disease; CKD = chronic kidney disease; ECMO = extracorporeal membrane oxygenation; APACHE II = Acute Physiology and Chronic Health Evaluation II.

Note:

1. Definitions of antiarrhythmic timing were not uniform across studies (e.g., arrest-to-drug, dispatch-to-drug, scene-arrival-to-drug, or timing relative to defibrillation); therefore, these values should not be interpreted as directly comparable across studies.
2. Definitions of witnessed arrest also varied across studies (e.g., any witnessed arrest, bystander-witnessed arrest, or EMS-witnessed arrest). Bystander CPR is principally applicable to OHCA and was therefore marked N/A for most IHCA studies.
3. Baseline severity was not measured using a uniform instrument and is summarized using the arrest- or illness-severity characteristics reported by each study, including rhythm status, CPR/arrest duration, comorbidity burden, refractory arrest, pre-arrest support, vasopressor use, and related clinical descriptors.
